# DIVAN: accurate identification of non-coding disease-specific risk variants using multi-omics profiles

**DOI:** 10.1186/s13059-016-1112-z

**Published:** 2016-12-06

**Authors:** Li Chen, Peng Jin, Zhaohui S. Qin

**Affiliations:** 1 Department of Mathematics and Computer Science, Emory University, Atlanta, GA 30322 USA; 2 Department of Human Genetics, Emory University School of Medicine, Atlanta, GA 30322 USA; 3 Department of Biostatistics and Bioinformatics, Rollins School of Public Health, Emory University, Atlanta, GA 30322 USA; 4 Department of Biomedical Informatics, Emory University School of Medicine, Atlanta, GA 30322 USA

**Keywords:** Non-coding variants, Disease-specific, Variant annotation, Epigenomics, Histone marks, Feature selection, Ensemble learning

## Abstract

Understanding the link between non-coding sequence variants, identified in genome-wide association studies, and the pathophysiology of complex diseases remains challenging due to a lack of annotations in non-coding regions. To overcome this, we developed DIVAN, a novel feature selection and ensemble learning framework, which identifies disease-specific risk variants by leveraging a comprehensive collection of genome-wide epigenomic profiles across cell types and factors, along with other static genomic features. DIVAN accurately and robustly recognizes non-coding disease-specific risk variants under multiple testing scenarios; among all the features, histone marks, especially those marks associated with repressed chromatin, are often more informative than others.

**Electronic supplementary material:**

The online version of this article (doi:10.1186/s13059-016-1112-z) contains supplementary material, which is available to authorized users.

## Background

With the development of high-density genotyping arrays, over the past ten years, investigators have conducted thousands of genome-wide association studies (GWAS), which have identified tens of thousands of loci associated with a host of human traits and diseases. There are now resources established to catalog a comprehensive collection of trait-associated single nucleotide polymorphisms (SNPs). One example, the Association Results Browser (ARB) (https://www.ncbi.nlm.nih.gov/projects/gapplus/sgap_plus.htm, accessed May 28, 2016) currently contains 44,124 SNP trait association results, which correspond to 30,553 (autosomes plus chromosome X) unique trait-associated SNPs linked to 573 phenotypes. Overall, 90% of those SNPs are located in non-coding regions (introns and intergenic regions), which is consistent with the observation that over 70% of the risk-association loci in the National Human Genome Research Institute (NHGRI) GWAS catalog lack variants that map to exons within their haplotype block [1].

Unlike coding variants, whose functional impact can be gauged by checking whether the DNA sequence variant affects the translated protein sequence [2], there is little we can say for non-coding variants, except about evolutionary conservation at the loci. Therefore, one needs information beyond the DNA sequence level to identify variants that functionally link to a disease or phenotype. Since non-coding SNPs are suspected of disrupting normal regulatory control mechanisms of target genes and we know that epigenetic information, such as DNase hypersensitivity and histone modifications, is closely related to regulatory function [3–5] and has been linked to the enrichment of GWAS SNPs [6], epigenetics data have thus been recognized as an important source of functional annotation for non-coding variants [3].

Taking advantage of the powerful high-throughput technologies, such as next-generation sequencing (NGS), experimental assays have been developed to comprehensively survey the entire genome for such regulatory events. Major experiments in this category include coupling chromatin immunoprecipitation and next-generation sequencing (ChIP-seq) [7–9] to identify in vivo binding of transcription factors (TFs) and histone marks; DNase I hypersensitive sites sequencing (DNase-seq) [10, 11] and formaldehyde-assisted isolation of regulatory elements sequencing (FAIRE-seq) [12], both for identifying open chromatin regions. Given the importance of such regulatory information, large international consortia, like the Encyclopedia of DNA Elements (ENCODE) [13] and the Roadmap Epigenomics Mapping Consortium (REMC) [14] have been formed to systematically conduct these experiments to identify functional elements with regulatory activities across hundreds of cell lines/tissues. These datasets offer a great opportunity to link sequence variants to regulatory elements, including TF binding, histone modification, and open chromatin.

Taking advantage of these resources, researchers have developed multiple computational approaches to identify non-coding risk variations. Ritchie et al. developed a supervised approach called Genome-Wide Annotation of Variants (GWAVA) [15], which is a modified random forest classifier [16], to distinguish disease-implicated variants from benign variants using various static genomic and epigenomic annotations, such as genic context, phylogenetic conservation scores, TF binding sites, and histone modifications. Kircher et al. developed a supervised learning approach named CADD [17], which is a support vector machine classifier that integrates 63 annotations, including phylogenetic conservation scores, genic context, and scaled *p* values derived from ENCODE, as features of the classifier. Lu et al. developed an EM-based algorithm called GenoCanyon [18] that models the non-coding variant using a two-component mixture model (risk or benign). Recently, Ionita-Laza et al. developed Eigen [19], another unsupervised approach adopting a more sophisticated two-component mixture model by imposing a predefined block-wise structure among features in the model-fitting process.

A common feature of all the above methods is that they are disease/phenotype neutral; that is, variants associated with all diseases/phenotypes are included in the training set. As an example, GWAVA uses all “regulatory mutations” from the public release of the Human Gene Mutation Database (HGMD) [20]. Eigen and CADD use GWAS index SNPs found in the US National Human Genome Research Institute’s GWAS catalog. GenoCanyon uses all the annotated variants from ClinVar [21]. However, it is likely that the biological functions underlying a risk variant for type 2 diabetes, a metabolic disorder, is different from that for Alzheimer’s disease, a neurodegenerative disorder. Furthermore, the regulatory activities of TFs and histone marks are different in different cell lines/tissues, sometimes dramatically, so it is not clear which combination of cell line/tissue and TFs/histone modifications could better distinguish risk variants of a particular disease/phenotype from benign variants. Therefore, we believe it is desirable and appropriate to develop a method that can identify disease-specific risk variants. This is particularly important for interpreting variants identified via personal genome sequencing (PGS), since most of the variants identified by PGS are rare variants (minor allele frequency less than 1%), making their association with disease difficult to measure using GWAS.

Here we present DIVAN (DIsease-specific Variant ANnotation), a novel method to identify disease-specific risk variants. DIVAN adopts an ensemble learning framework with a feature selection step to annotate and prioritize non-coding variants using a large collection of genomic and epigenomic annotations. To evaluate DIVAN’s performance, we conduct comprehensive analyses using data from two different databases. One study involves 45 different diseases/phenotypes across 12 disease/phenotype classes and the other includes 36 diseases/phenotypes.

In this work, we treat the trait-associated index SNPs identified by GWAS and reported in the ARB as surrogates for the functional SNPs. This is because validated or annotated bona fide functional SNPs are too rare for most diseases/phenotypes to form a meaningful training set. Furthermore, the belief is that real functional variants are enriched among GWAS index SNPs than random background SNPs.

## Results

### Overview of the DIVAN approach

**Fig. 1:**
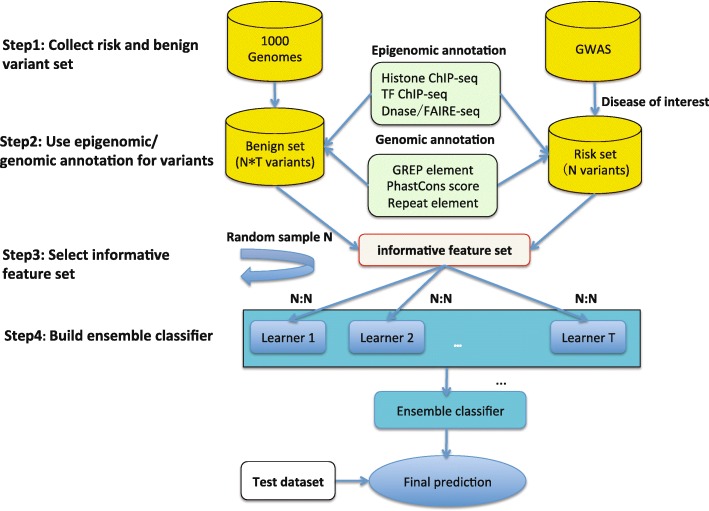
*Flow chart* for the DIVAN approach

The main challenge to disease-specific variant annotation is that the size of the training set is often small as the disease-specific risk variants identified by GWAS with high confidence (stringent *p* values) is often very limited as the median of trait-SNP associations is only 8 for the 573 traits in the ARB. On the other hand, to improve predictive performance, we attempt to include as many genome-wide genomic and epigenomic features as possible, often thousands of them (made possible given the abundant TFs/histone modifications across many cell lines/tissues), resulting in a typical “large *p*, small *n*” problem [22]. Thus, simply fitting the predictive model with all features would easily cause over-fitting. To accommodate as many features as possible while avoiding over-fitting, we employ two important machine learning strategies in DIVAN: feature selection and ensemble learning [23]. Feature selection is used to select the informative set of features that contribute most to the predictive performance and ensemble learning enables better predictive performance by creating a balanced risk/benign variant set in each base learner. The entire procedure of DIVAN is illustrated in Fig. 1.

#### Diseases studied

We conduct extensive real data analyses to evaluate the performance of DIVAN in detecting disease-specific risk variants. Out of the total of 573 diseases/traits found in ARB, 45 of them, spanning 12 disease classes, contain at least 50 reported disease-SNP associations. These diseases are included in our study. A complete list of the diseases/phenotypes along with the number of associated risk variants are summarized in Additional file 1: Table S1.

#### Features considered

As shown in Table 1, we use 1806 epigenomic features in this study, including features related to histone modification (1002), TF binding (571), open chromatin (184), and RNA Pol II/III binding (49), spanning 261 cell lines. Features are represented by read counts in the neighborhoods of each variant and reads from biological replicates (same factor and same cell line) are further merged. More detailed descriptions of these features can be found in the “Methods” section.

**Table 1:** Summary of feature categories in DIVAN

Data source	Cell lines	Factors	Features
REMC DNase	73	-	73
REMC Histone	109	31	735
ENCODE DNase	80	-	80
ENCODE FAIRE	31	-	31
ENCODE TF(HAIB)	19	76	292
ENCODE TF(SYDH)	31	100	279
ENCODE Histone	18	42	267
ENCODE RNA Polymerase	31	2	49
Total	261^a^	217^a^	1806

^a^The same cell lines or factors may appear in multiple sources

### Characteristics of epigenomic profiles around risk variants

**Fig. 2:**
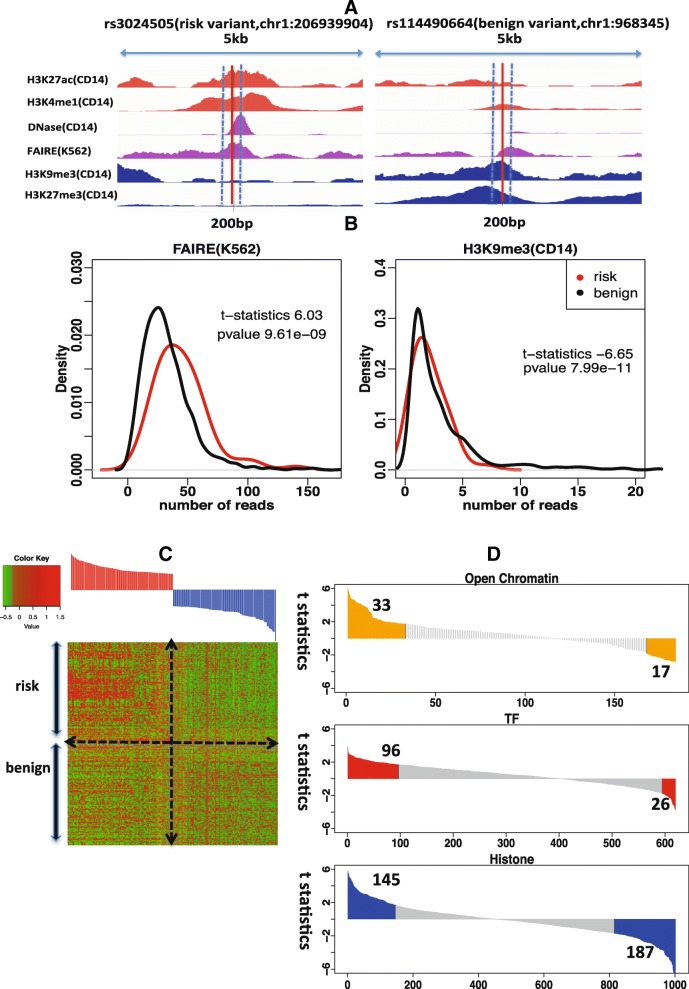
Epigenomic profiles of risk variants and benign variants. **a** Epigenomic profiles of active chromatin marks, H3K27ac and H3K4me1, repressive chromatin marks, H3K9me3 and H3K27me3, open chromatin regions in the CD14 and K562 cell lines in the neighborhoods of a risk variant, rs3024505 (chr1:206939904), associated with type 1 diabetes, and a benign variant, rs114490664 (chr1:968345). **b** Distribution of read counts for FAIRE-seq in the K562 cell line across 147 risk variants associated with type 1 diabetes and corresponding benign variants; distribution of read counts of H3K9me3 ChIP-seq in the CD14 cell line across 147 risk variants associated with type 1 diabetes and corresponding benign variants. **c** *Heatmap* of standardized read counts of top 100 epigenomic features and bottom 100 epigenomic features across 147 risk variants associated with type 1 diabetes and 147 corresponding benign risk variants. Epigenomic features are ranked by the t-statistics from the most enriched to the most depleted in risk variants compared to benign variants. Read counts are standardized by subtracting the average of read counts of each feature and divided by the standard deviation of read counts of each feature. **d** Distribution of t-statistics for three types of epigenomic features: TF binding, histone modification, and open chromatin. Within the informative features, 33 informative open chromatin-associated features are enriched while 17 informative open chromatin-associated features are depleted; 96 TF-associated informative features are enriched while 26 TF-associated informative features are depleted; 145 informative histone-associated features are enriched while 187 informative histone-associated features are depleted

Open chromatin regions marked by selected histone marks or DNA hypersensitivity are known to harbor GWAS risk variants [14]. For demonstration purposes, we present the sequencing read abundance pattern of selected epigenomic marks in the neighborhoods of a type 1 diabetes-associated risk variant (rs3024505) and a benign variant (rs114490664) on chromosome 1 (Fig. 2a). One can see that the neighborhoods of risk variant rs3024505 are enriched in the active chromatin marks, H3K27ac and H3K4me1, as well as an open chromatin regions defined by DNase-seq and FAIRE-seq in the CD14 or K562 cell line. In contrast, repressive chromatin marks, such as H3K9me3 and H3K27me3 in the CD14 cell line, are depleted around risk variant rs3024505 versus benign variant rs114490664.

We further investigate whether some epigenomic features differ in terms of the distribution of neighborhood read counts between risk variants and benign ones. Those epigenomic features showing a significant distribution difference are considered informative features. As an example, FAIRE-seq in the K562 cell line shows significant read enrichment (t-test statistics 6.03, *p* value < 10^–8^) around risk variants associated with type 1 diabetes compared to benign ones, while H3K9me3 in the CD14 cell line shows significant read depletion around risk variants (t-test statistics –6.65, *p* value < 10^–10^) (Fig. 2b).

For illustration purposes, Fig. 2c shows 200 epigenomic profiles represented by read counts in the neighborhoods of 147 risk variants associated with type 1 diabetes and 147 randomly selected benign variants. The top 100 features that are mostly enriched in risk variants compared to benign ones and the bottom 100 features that are mostly depleted in risk variants. Clearly, there exist different enrichment patterns for the two sets of variants in these selected features.

For the informative features with *p* values of t-test below 0.09 (0.09 is the selected *p* value threshold for type 1 diabetes using the method described in the “Methods” section), we find that more features associated with open chromatin or TF binding show enrichment around risk variants, while more features associated with histone modifications show depletion around risk variants (Fig. 2d). As type 1 diabetes is an immune-related disease, it is interesting to observe that all eight features associated with open chromatin in the cluster of differentiation (CD) cell line show enrichment in risk variants, while 14 features associated with H3K9me3 in the CD cell line show depletion.

### Performance evaluation

To evaluate the performance of DIVAN, we compare DIVAN with four different risk variant annotation and prioritization methods: GWAVA, CADD, Eigen, and GenoCanyon.

### Disease-specific variant prioritization evaluation using cross-validation

**Fig. 3:**
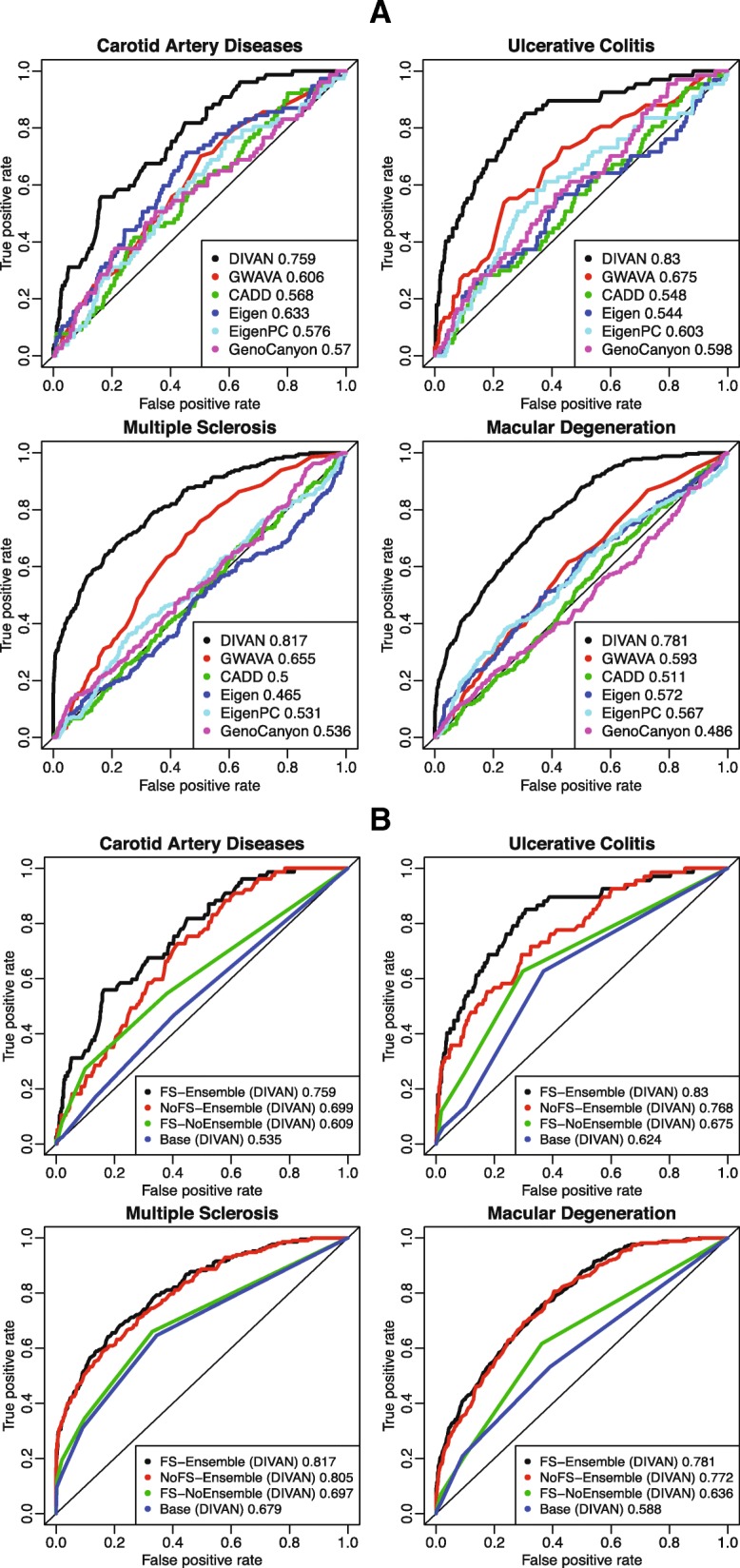
Predictive performance of fivefold cross-validation on four diseases: carotid artery disease, macular degeneration, ulcerative colitis, and multiple sclerosis. **a** *ROC curves* comparing the predictive performance among DIVAN and CADD, GWAVA, Eigen, Eigen-PC, and GenoCanyon for the four diseases. **b** *ROC curves* showing the effectiveness of feature selection and ensemble method by comparing feature selection and ensemble combined, feature selection only, ensemble only, and the baseline case: neither feature selection nor ensemble

**Fig. 4:**
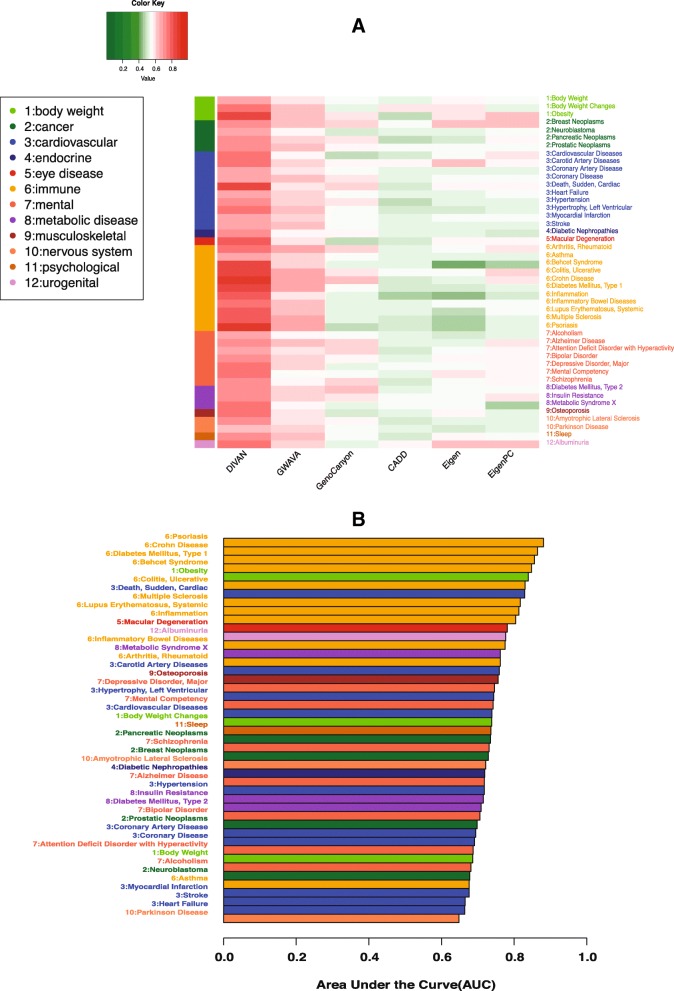
Predictive performance of fivefold cross-validation across 45 diseases in 12 disease classes. **a** *Heatmap* of fivefold cross-validation AUC values for predictive performance comparison among DIVAN and CADD, GWAVA, Eigen, Eigen-PC, and GenoCanyon across 45 diseases in 12 disease classes. **b** *Bar charts* of fivefold cross-validation AUC values of DIVAN across 45 diseases in 12 disease classes ranked in decreasing order. Disease classes are color-coded

Fivefold cross-validation is used to evaluate the predictive performance of different methods, and results are presented in the form of receiver operator characteristics (ROC) curves with corresponding area under the curve (AUC) values. For demonstration purposes, we present here results from four diseases: carotid artery disease (cardiovascular disease), macular degeneration (eye disease), ulcerative colitis (digestive system disease/immune disease), and multiple sclerosis (immune disease) in Fig. 3a. Additional file 2: Figure S1 shows the corresponding precision recall curves for the four diseases. The remaining 41 ROC curves are presented in Additional file 2: Figure S2. Overall, DIVAN achieves the best predictive performance among all methods, with AUC values in the range of 0.65–0.88 (median 0.74), followed by GWAVA and GenoCanyon. For a comprehensive comparison, we present the AUC values of all methods compared across 45 diseases in a heatmap (Fig. 4a). The AUC values are included in Additional file 1: Table S2 and the average Matthews correlation coefficient (MCC) values of different methods across 45 diseases are shown in Additional file 1: Table S3. Moreover, we find DIVAN performs the best among immune-related diseases, followed by multiple eye diseases and urogenital disorders. On the other hand, identifying risk variants associated with mental disorders and cardiovascular diseases seems more challenging for DIVAN (Fig. 4b).

### Disease specificity of variant annotation

A key feature of DIVAN lies on its disease-specificity, which means the predictive model is trained disease by disease using annotated disease-specific variants. To justify the necessity of the disease-specific assumption, we conduct an experiment in which a model trained using variants from one disease is subsequently applied to classify variants annotated for a different disease. In the experiment, we use four diseases from distinct disease classes: carotid artery disease (cardiovascular disease), macular degeneration (eye disease), Alzheimer’s disease (mental disease), and multiple sclerosis (immune disease). For the same disease training and testing, we report the AUC values of fivefold cross-validation. As expected, we find decreased AUC values when a model trained in one disease is applied to a different disease (Additional file 2: Figure S3A, B), which confirms the advantage of using the disease-specific model adopted by DIVAN.

### Effectiveness of feature selection and ensemble learning

To demonstrate the effectiveness of adopting the feature selection and ensemble learning strategies, we conduct a performance comparison using four different settings: baseline (no feature selection, no ensemble learning), feature selection only, ensemble learning only, and feature selection combined with ensemble learning. Again, we use the four aforementioned diseases as representatives and fivefold cross-validation to evaluate the predictive performance and results are presented in the form of ROC curves with corresponding AUC values (Fig. 3b), as well as precision recall curves (Additional file 2: Figure S4). The results confirm that feature selection combined with ensemble learning achieves the best performance. Moreover, either feature selection or ensemble learning alone improves the predictive performance compared to the baseline.

### Contribution of different feature groups

Since most epigenomic features used in DIVAN come from three groups—TF binding, histone modifications, as well as open chromatin (DNase-seq and FAIRE-seq)—it would be interesting to investigate which feature group contributes relatively more to risk variant identification. In addition, existing methods use called peaks from sequencing-based assays to represent epigenomics features, which is a binary indicator of whether a variant overlaps with any peak (referred to as peak hereafter). Instead, by default DIVAN uses read counts in the neighborhood of the variant as the feature representation (referred to as read hereafter) for the robustness of predictive performance when limited features are available.

To compare performance with different feature groups and different feature representations, we apply DIVAN to the aforementioned four diseases in different settings. We find that no matter whether peak or read is used, using all feature groups achieves the best performance, as expected; and using features related to histone modifications alone could achieve better predictive performance than any other feature group. However, the contribution of each feature group when using peak and read differs slightly (Additional file 2: Figure S5A, B). Specifically, using features related to histone modifications alone achieves comparable predictive performance no matter whether peak or read is used, whereas using read shows much better performance than using peak as the feature representation for TF binding and open chromatin. A possible explanation is that the continuous read counts are more sensitive than peak overlap in detecting subtle differences between risk and benign variants, especially when genome-wide coverage of the feature is relatively sparse, such as TF binding or open chromatin.

### Disease-class variant prioritization

Diseases/phenotypes in the same disease/phenotype class are believed to be likely more phenotypically related to each other and we want to investigate the predictive performance when including risk variants from diseases/phenotypes that belong to the same class into the training set. This strategy is called disease-class specificity, which is an extension of the disease-specificity strategy adopted so far. Because only a handful of risk variants have been identified by GWAS for most of the diseases/phenotypes, this strategy is rather attractive since it allows the critically needed boost to the training set when only a few variants have been identified.

To demonstrate the utility of this assumption, we perform a “leave-one-disease-out” testing approach; that is, we build the model using known risk variants of all but one disease within the disease class and apply the model to identify risk variants for the omitted disease. To illustrate the performance of this strategy, we take five immune diseases reported in ARB, including rheumatoid arthritis, asthma, type 1 diabetes mellitus, systemic lupus erythematosus, and multiple sclerosis, as examples. We observe promising predictive performance since all AUC values are above 0.8, except for asthma (Additional file 2: Figure S6).

### Applying DIVAN to disease-specific variants in the GRASP database

To further evaluate the performance of DIVAN, we take on a different testing set using risk variants in the GRASP database [24], which includes around 8.87 million SNPs identified from 2082 GWAS (accessed Mar 30, 2016). The large size of the database is mainly due to the fact that a less stringent *p* value threshold (0.05) is used for risk SNP inclusion. For the testing set, we are able to match 36 out of 45 ARB diseases in GRASP; for each disease, we only keep risk variants in non-coding regions with a *p* value less than 10^–4^ and further exclude risk variants collected in ARB for the same disease/phenotype; we further remove duplicated variants (the same SNP being reported multiples times from different platforms or different studies) in GRASP.

The corresponding benign variants are selected by randomly sampling ten times the number of risk variants of each disease from the catalog of the 1000 Genomes Project, excluding all GRASP variants.

**Fig. 5:**
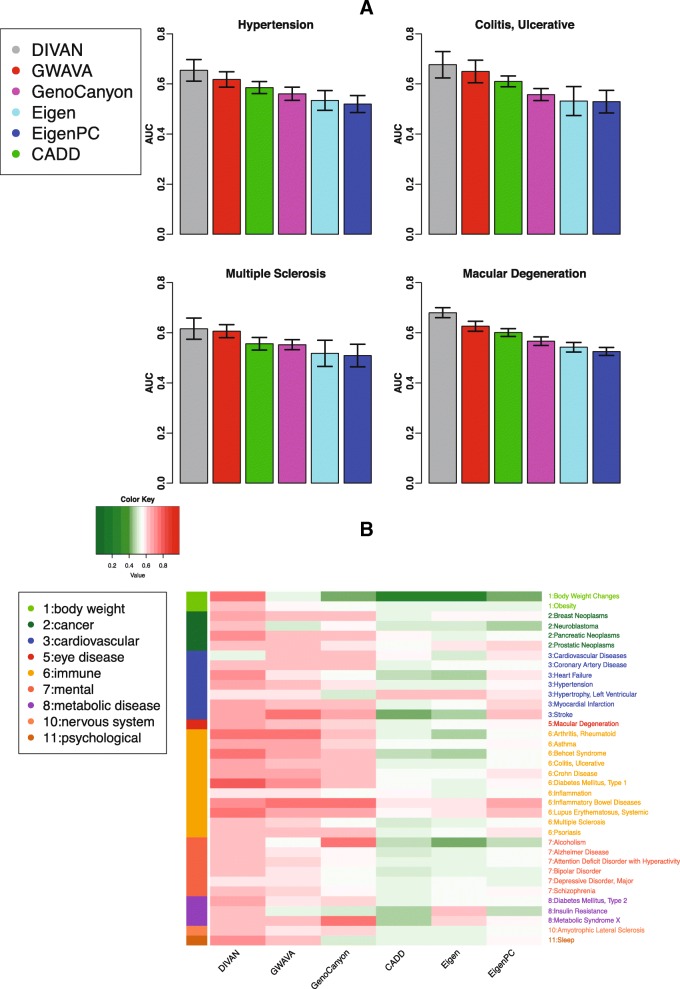
Predictive performance on 36 diseases in GRASP database. **a** *Bar charts* of AUC values among DIVAN and CADD, GWAVA, Eigen, Eigen-PC, and GenoCanyon for four diseases: hypertension, macular degeneration, ulcerative colitis, and multiple sclerosis. The *bar charts* are sorted by the mean AUC values and the *error bar* describes the standard deviation. The training set is risk variants collected from the ARB and the testing set is the risk variants collected from GRASP. **b** *Heatmap* of mean AUC values for predictive performance comparison among DIVAN and CADD, GWAVA, Eigen, Eigen-PC, and GenoCanyon across 36 diseases investigated

For each of the 36 diseases, we use the same set of risk variants in ARB as the training set and the risk variants in GRASP but not in ARB as the testing set. The number of training and testing variants for the 36 diseases are summarized in Additional file 1: Table S1. To avoid possible bias due to sampling variability, for each disease, we repeat the whole procedure ten times with a different set of benign variants (by random sampling) each time and calculate the average AUC values. For illustration purposes, we compare the AUC values of different methods for the four representative diseases (Fig. 5a). DIVAN shows the highest AUC values once again. For an overview, we present the average AUC values of different methods across all 36 diseases in a heatmap (Fig. 5b) and in Additional file 1: Table S4, and the average MCC values of different methods across 36 diseases are shown in Additional file 1: Table S5. Overall, DIVAN shows the best performance as it achieves the highest AUC values in 27 out of 36 diseases and is close to the best in the remaining nine diseases. GWAVA has the second-best predictive performance for obtaining the highest AUC values in four diseases, followed by GenoCanyon, with the highest AUC values in three diseases. For AUC values achieved by DIVAN, we find it performs the best for immune-related diseases, which is consistent with the findings from the 45 ARB diseases using fivefold cross-validation.

### Applying DIVAN to regulatory variants in the HGMD database

So far the risk variants we use are collected from ARB and GRASP databases where the variants are disease-implicated by GWAS. It is also of great interest to test variants from other sources. There are well-known databases available that contain curated variants, which are often carefully selected by experts. For example, variants with pathogenic or non-pathogenic effects in ClinVar are collected from literature evaluation, clinical testing and research, and reviewed by different expert groups. Mutations in HGMD are collected from the literature. Unfortunately, among the collected 194 non-coding ClinVar variants used by GWAVA, none of them are associated with any of the 45 diseases in ARB used in the training set. This might be attributed to the fact that the majority of the variants in ClinVar are either coding variants or associated with Mendelian diseases. Because DIVAN is disease-specific and requires training and testing set from the same disease, we choose not to test DIVAN on ClinVar variants.

For HGMD, we collect 1614 disease-associated regulatory variants used by GWAVA. In order to find out which disease is associated with each variant, we manually query each of the 45 diseases on HGMD website to retrieve all regulatory variants in HGMD that are associated with any of the 45 diseases. By looking for the overlap between the two sets of variants, we identify 117 unique autosomal variants (excluding sex chromosomes and mitochondria) associated with at least one of the 45 diseases.

Among these 117 variants, very few of them (less than 15) map to any one of the 45 diseases individually, which is not enough to get meaningful comparison results for a disease-specific study. Fortunately, we find that there are 34 variants associated with at least one disease in the immune disease class including Asthma, Behcet syndrome, Ulcerative colitis, Crohn’s disease, Inflammatory bowel diseases, and Systemic lupus erythematosus. Hence, we group the 34 variants associated with diseases in the immune disease class as an independent testing set, conduct a disease class-specific analysis using DIVAN, and compare the predictive performance with other methods. The corresponding benign variants of the 34 immune disease-associated variants in the testing set are chosen in the same way as for the GRASP testing set. For this experiment, we do not include GWAVA since it uses the 1614 HGMD variants as its training set. For DIVAN, we train a disease class-specific model by pooling all the variants in ARB that are associated with any of the aforementioned six immune-related diseases together in the training set. For other methods that are not disease-specific, we use their pre-computed scores. The AUC values are summarized in Additional file 1: Table S6. There we see that DIVAN virtually tied with GenoCanyon and is better than CADD, Eigen, and EigenPC. The results demonstrate DIVAN’s robust performance on different independent testing sets.

### Applying DIVAN on synonymous mutations

Though DIVAN is designed for the identification of non-coding variants, it is interesting to see how DIVAN performs on coding variants especially synonymous mutations.

We collect synonymous mutations from the online database dbDSM [25], which is a manually curated database that collects 1936 synonymous mutations-disease association entries. In total, we have 1109 autosomal synonymous mutations (excluding sex chromosomes and mitochondria). We find seven diseases associated with more than 20 synonymous mutations in dbDSM are also among 45 diseases in ARB; hence, we use the seven diseases for performance comparison. The corresponding benign variants for each disease in the testing set are chosen in the same way as for GRASP testing set. The AUC values are reported in Additional file 1: Table S7.

The results show that overall GWAVA performs the best while DIVAN is on par with the other methods, suggesting DIVAN is not as good in predicting coding variants as it predicts non-coding variants. This is not surprising since all the features and the training procedure used by DIVAN are optimized for prioritizing non-coding variants. On the other hand, GWAVA uses HGMD regulatory mutations as the training set in which 75% of them lies within a 2 kb window around transcription start site (TSS), indicating the majority of HGMD mutations is close to the coding regions. That might explain the better performance of GWAVA. In the future, we plan to extend DIVAN’s functionality to identify disease-specific coding variants, by perhaps adding coding-region specific features.

### Exploration and interpretation of features

#### Variability of factors across cell types

A key merit of DIVAN is its ability to consider a large number of cell type-specific epigenomic profiles as features to accommodate the cell type-specific nature of the epigenome, which aims to include as many features as possible, without any screening upfront, and let the algorithm select informative features automatically. For some existing methods, such as GenoCanyon and Eigen, epigenomic profiles of the same factor across different cell types are collapsed to simplify the model or speed up computation. That way, the plastic epigenomic profiles across cell types are ignored.

**Fig. 6:**
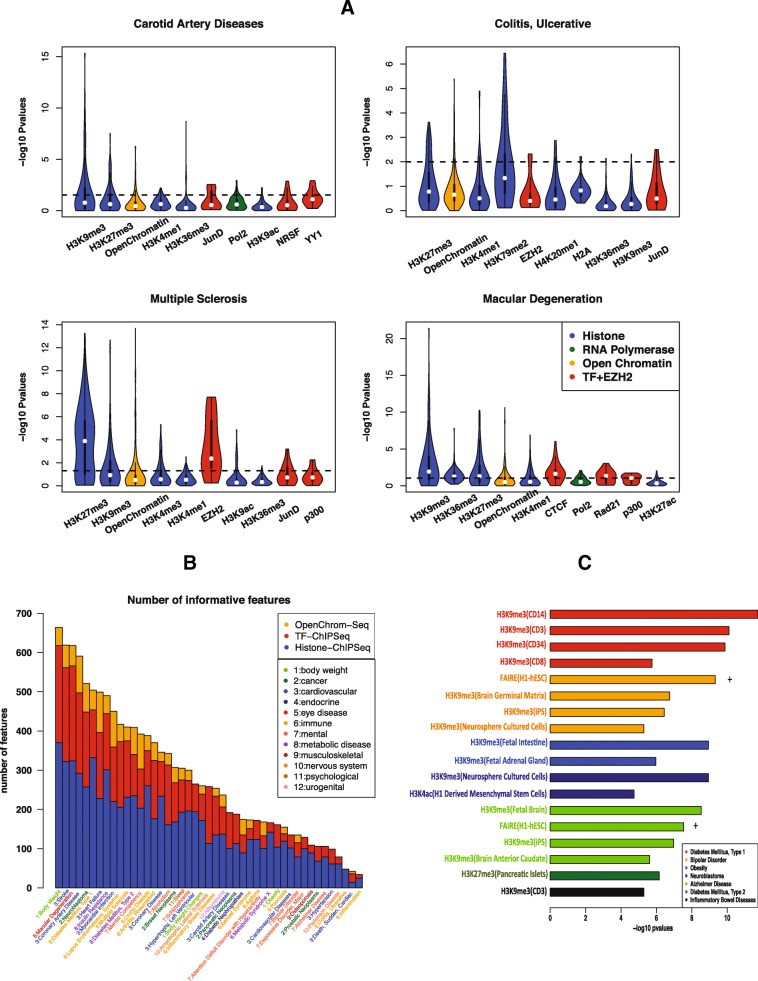
Exploration and interpretation of epigenomic features. **a** *Violin plot* for the distribution of –log10 *p* values of the top ten factors (TF binding, histone modification, open chromatin, RNA polymerase) associated with more than ten epigenomic features for four diseases: carotid artery disease, macular degeneration, ulcerative colitis, and multiple sclerosis. *P* values are calculated by t-test on the read counts in the neighborhoods of the risk variants and benign variants. **b** Number of informative features for three feature categories (TF binding, histone modification, open chromatin) for 45 diseases across 12 disease classes using read as the feature value. **c** *Bar chart* of –log10 *p* values for top-ranked features for selected diseases: type 1/type 2 diabetes, bipolar disorder, obesity, neuroblastoma, Alzheimer’s disease, and inflammatory bowel disease

To show the variability of epigenomic factors across cell types and the dynamic profiles of epigenomic factors across diseases, we obtain the *p* values from t-tests conducted between the risk and benign variants across 1806 epigenomic features for the four aforementioned diseases. We sort the factors profiled in more than ten cell types by the number of features remaining in the informative feature set and plot the log-transformed *p* values (Fig. 6a). One can see that there is considerable variability of the *p* values for the same factor across different cell types, which confirms the necessity of considering the combination of factors and cell types as the epigenomic features. Moreover, the rank of factors varies from disease to disease, further reflecting the variable nature of these factors.

Overall, we see that the top-ranked factors for the four diseases are two repressive chromatin marks, H3K9me3 and H3K27me3, followed by open chromatin, and two active chromatin marks, H3K4me1 and H3K36me3. The top factor is H3K9me3 for carotid artery disease and macular degeneration and H3K27me3 for ulcerative colitis and multiple sclerosis. Both factors are repressive chromatin marks. JunD, Pol2, and p300 also frequently rank high. On the other hand, active chromatin marks, e.g., H3K4me3 and H3K27ac, do not always appear among the top factors. Moreover, it is interesting to see that EZH2 and H3K27me3 both top rank in multiple sclerosis and ulcerative colitis as EZH2 represses gene transcription by mediating H3K27me3 methylation [26].

### Informative features across different diseases

As the informative feature set helps improve the predictive performance, we further investigate the number of informative features selected within three feature groups: histone modification, TF binding, and open chromatin (Fig. 6b). Overall, the total numbers of informative features selected vary from disease to disease, ranging from 664 (body weight) to 34 (inflammation) if read is used as the feature, while the overall numbers of informative features decrease, ranging from 549 (type 2 diabetes) to 41 (obesity) if peak is used as the feature (Additional file 2: Figure S7).

We also observe that the histone modifications feature group contributes more to informative features than the TF binding or open chromatin feature group. Moreover, more TF-associated and fewer histone-associated features show up in the informative feature set when read rather than peak is used as the feature (Fig. 6b and Additional file 2: Figure S7).

### Interpretation of top features

Although the main goal of DIVAN is to distinguish disease-specific risk variants from the vast pool of benign ones, we demonstrate that the feature selection step could also help identify top features that are biologically meaningful.

To illustrate, we present some of the top features identified from selected diseases and the observed enrichment/depletion patterns are readily interpretable (Fig. 6c). For example, we find that H3K9me3 in CD cells, known to be on the cell lineage that leads to immune-related disease, is depleted around the risk variants associated with type 1 diabetes. Interestingly, H3K9me3 in CD cells is also depleted around risk variants associated with another immune-related disease: inflammatory bowel disease. H3K27me3, another repressive chromatin mark, in pancreatic islet cells is found to be depleted around risk variants associated with type 2 diabetes, a disease caused by pancreatic islet dysfunction. For bipolar disorder, we find open chromatin regions in H1 cells measured by FAIRE-seq are enriched, while H3K9me3 in the brain germinal matrix, iPS, and neurosphere cultured cells is depleted in the neighborhoods of their risk variants. Risk variants associated with another mental disorder, Alzheimer’s disease, are also depleted of H3K9me3 in fetal brain, iPS, and brain anterior caudate cells, but enriched of open chromatin regions in H1 cells measured by FAIRE-seq. Risk variants associated with obesity are depleted of H3K9me3 in fetal intestine and fetal adrenal gland cells. H3K9me3 in neurosphere cultured cells and H3K4ac in H1-derived mesenchymal stem cells are depleted around risk variants associated with neoplasms. For the above diseases investigated, we find that H3K9me3 consistently shows depletion, while open chromatin consistently shows enrichment around risk variants.

### H3K9me3 is the most informative factor for risk variant identification across diseases/phenotypes

In addition to identify informative epigenomic factors for differentiating risk variants from benign variants in each individual disease, we also want to identify the “frequent fliers,” i.e., the epigenomic factors that contribute to a wide spectrum of diseases. To find out, for each disease, we check which factors are over-represented in the list of identified informative features using a binomial test. Let *n*_*i*_ represent the number of informative features in disease *i*; *N* the total number of features in this study (1806); *m*_*ij*_ represent the number of features associated with factor *j* in disease *i*; *k*_*ij*_ represent the number of informative features associated with factor *j* in disease *i*. The *p* value for factor *j* over-represented in disease *i* could be calculated as:

$$ \begin{array}{l}p\left(x>{k}_{ij}\Big|{n}_i,{p}_{ij}\right)={\displaystyle \sum_{x={k}_{ij}+1}^{n_i}}\left(\begin{array}{c}\hfill {n}_i\hfill \\ {}\hfill x\hfill \end{array}\right){{\widehat{p}}_{ij}}^x{\left(1-{\widehat{p}}_{ij}\right)}^{n_i-x}\\ {}{\widehat{p}}_{ij}=\frac{n_i}{N\ }\end{array} $$

**Fig. 7:**
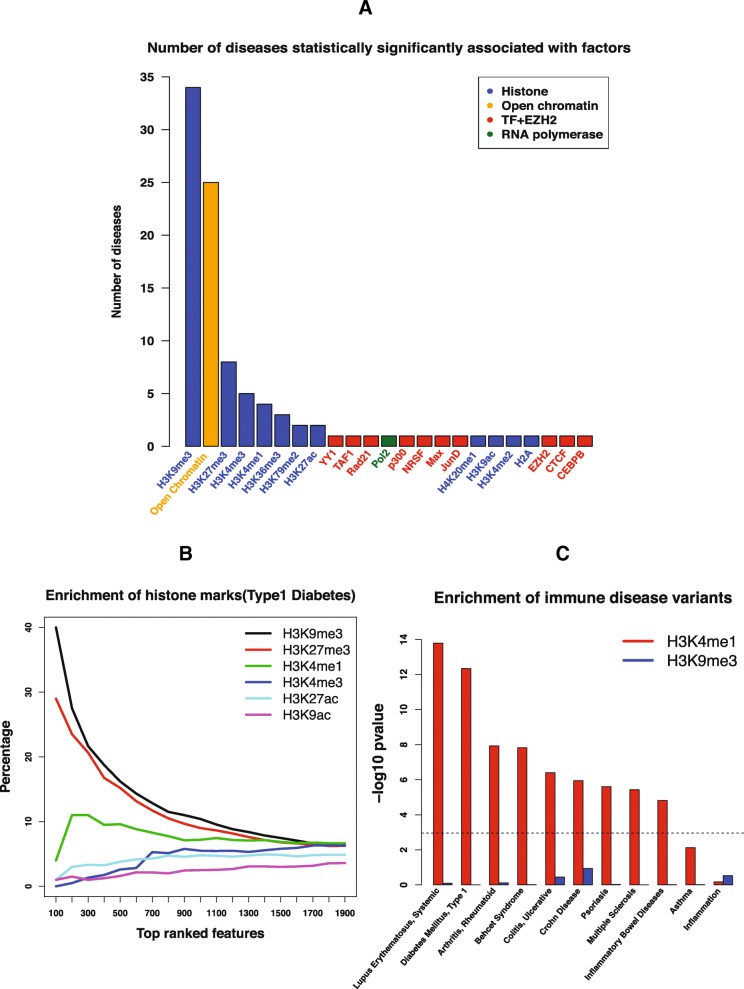
Association between factors and diseases. **a** Number of diseases statistically significantly associated with different factors (TF binding, histone modification, open chromatin, RNA polymerase). **b** Enrichment of different histone marks among top features for type1 diabetes. **c** Enrichment of risk variants associated with immune disease in peaks of active chromatin mark H3K4me1 in the CD14 cell line and peaks of repressive chromatin mark H3K9me3 in the CD14 cell line

Any factor with *p* value less than the Bonferroni corrected threshold (0.05/45) is said to be over-represented in the disease *i*. At the end, for each factor, we tally the number of times it is over-represented across all 45 diseases (Fig. 7a). We find that H3K9me3 and open chromatin are the top informative factors; H3K9me3 is over-represented in 34 out of 45 diseases, while open chromatin is over-represented in 25 out of 45 diseases.

Consistent with previous findings that histone marks are the most frequent features to be ranked at the top among the three types of epigenomic features (Fig. 6b), Fig. 7a shows that histone marks are associated with more diseases than TFs overall; however, to our surprise, among the histone marks that are most significant, most of them are associated with repressive chromatin, such as H3K9me3 and H3K27me3, and H3K9me3 in particular. We also confirm the well-documented fact that open chromatin marked by DNase-seq and FAIRE-seq is enriched around risk variants [27].

To further illustrate the dominance of H3K9me3 compared to other histone marks among top features, we plot the enrichment of different histone marks sorted by *p* values for type 1 diabetes (Fig. 7b). H3K9me3 is the most over-represented factor among the informative features, associated with 40% of the top 100 features, followed by H3K27me3 (29%) and H3K4me1 (4%). Other marks associated with active chromatin, H3K4me3, H3K27ac, and H3K9ac, are not significantly enriched among the top features.

It has been shown that genomic regions marked by active chromatin, such as H3K4me1, are enriched near GWAS-identified risk variants [14, 28], so we are interested to see whether regions marked by repressive chromatin, such as H3K9me3, are depleted by risk variants. To do this, we collect the called peaks of H3K9me3 and H3K4me1 in the CD14 cell line, known to be from the cell lineage that leads to immune-related diseases, and calculate the enrichment of risk variants associated with each of the 45 diseases in those peaks using traseR [28], an R package that is capable of searching and ranking diseases/phenotypes for a given set of genomic regions based on the enrichment level of trait-associated SNPs. We plot the *p* values on the logarithm scale of the enrichment test across 11 immune diseases (Fig. 7c). We find that none of the immune-related diseases are statistically significantly enriched in H3K9me3, while all but asthma and inflammation are statistically significantly enriched in H3K4me1.

### Additional tests on more settings of DIVAN

For a complex machine learning problem like the one we are tackling, different settings in training and testing might cause overestimate or underestimate of the actual performance. Here we carry out additional tests under different experimental settings to investigate the robustness of DIVAN’s performance.

### Different sources of benign variants in the training set

Currently, the set of benign variants are chosen from the 1000 Genomes (phase I). Since the risk variants are mostly GWAS SNPs, to avoid picking up features that might be a by-product of SNP design and selection, we instead choose benign variants from GWAS SNPs as well, found on one of the latest GWAS genotyping array—Affymetrix Genome-Wide Human SNP array 6.0. To be specific, we collect 900,611 non-coding GWAS SNPs out of 934,968 GWAS SNPs from the SNP annotation file to construct the set of benign variants for each disease. Using the new set of benign variants, we retrain the disease-specific model for the 45 diseases in ARB, obtain the CV-AUC values (Additional file 1: Table S8) for the fivefold cross-validation and the predicted AUC values for the 36 diseases in GRASP in the independent test (Additional file 1: Table S9 and S10).

For the 45 diseases found in ARB, the Pearson correlation coefficient between the two sets of AUC values is 0.979 (*p* value < 2.2e-16). The average CV-AUC values for the 45 diseases changes from 0.745 (sd: 0.060) to 0.742 (sd: 0.061). For the 36 diseases found in GRASP, the Pearson correlation coefficient between the two sets of AUC values is 0.950 (*p* value < 2.2e-16). The average predicted AUC values for the 36 diseases changes from 0.661 (sd: 0.055) to 0.658 (sd: 0.061). The results show that the AUC values are similar either using SNPs from the GWAS genotyping array or using SNPs 1000 Genomes to form the set of benign variants in the construction of disease-specific model.

### Different criteria of choosing benign variants in the training set

By default, DIVAN uses distance to the nearest TSS as the criterion to choose a set of benign variants such that distances to the nearest TSS matched (have a similar empirical distribution) with those of the risk variants. The distance to TSS-matched criterion keeps the two sets (risk and benign) on leveled grounds in their chromatin profiles because non-coding disease-associated variants in ARB tend to locate close to TSS (mostly within 200 kb, Additional file 2: Figure S8) and chromatin landscape is quite different between promoter regions and intergenic regions. The same criterion has also been adopted by GWAVA.

We also adopt an alternative and perhaps more stringent criterion to choose the set of benign variants in the training set in which we require that all benign variants have to be located within 10 kb of a risk variant. Here we use a slightly wider region than the 1 kb region used by GWAVA but narrower than the 100 kb region used by Eigen. This is because the histone mark profiles, which DIVAN used predominantly, typically extend to a few kbs.

We conduct another test using the new region-matched benign set (denoted as region) and compare the results with the results obtained earlier using the distance to the TSS-matched benign set (denoted as TSS). We find average CV-AUC values for the 45 diseases in ARB changes from 0.745 (sd: 0.060) to 0.680 (sd: 0.037) and the average AUC values for the 36 diseases in GRASP changes from 0.661 (sd: 0.055) to 0.637 (sd: 0.043). The CV-AUC values are shown in Additional file 1: Table S11. The decrease of predictive performance using the region-matched benign set is consistent with what is observed in GWAVA. Despite the slight drop in performance when using the region-matched criterion, DIVAN still maintains its lead over all the competitors tested. In the independent test, among the 36 diseases in GRASP, DIVAN is the best performer in 23 diseases, followed by GWAVA (seven diseases), GenoCanyon (four diseases), and Eigen (two diseases). The predicted AUC values are shown in Additional file 1: Table S12 and S13.

### Impact of nearby variants on cross-validation

In the cross-validating study described earlier, although there is no overlap of variants between the training and the testing sets, it is possible that a risk variant in the testing set is located near a risk variant in the training set which may potentially inflate the CV performance. In order to eliminate such influence, before preforming CV, we further remove risk variants that are too close to each other and do the same thing for benign variants as well. To be specific, we first sort all risk variants (or benign variants) based on their genomic locations and only keep one variant if multiple variants happen to be less than 10 kb away. That way, we make sure that neither training folds nor the testing fold contains risk variants (or benign variants) at the same or nearby location (10 kb). The updated numbers of risk variants for the 45 diseases in ARB are shown in Additional file 1: Table S1. The numbers of risk variants of the 45 diseases in ARB decrease around 16% on average.

To evaluate the impact of this change, we conduct an experiment using the new rule and compare the results with those obtained before. We retrain all the disease-specific models and calculate the CV-AUC values for the 45 diseases in ARB (Additional file 1: Table S14). We find that using the new rule, the average AUC values for the 45 diseases changes from 0.745 (sd: 0.060) to 0.736 (sd: 0.056) and the Pearson correlation coefficient between the two sets of CV-AUC values is 0.917 (*p* value < 2.2e-16). In conclusion, we see little difference the new rule has on the outcome of CV-AUC values. DIVAN still outperforms all the competitors by a comfort margin.

### Impact of nearby variants on independent test

For the independent test described earlier, although we have excluded all ARB variants from the GRASP testing set, it is possible that some variants in the GRASP testing set are located near ARB variants used in the training set, which may affect the independent test performance. Therefore, to eliminate such influence, for each disease, we further exclude risk variants in the GRASP testing set that are close to risk variants found in the ARB training set. The updated numbers of disease-associated SNPs for the 36 diseases in GRASP can be found in Additional file 1: Table S1. The numbers of risk variants of 36 diseases in GRASP decrease around 7% on average.

To be specific, for each disease, hypertension for example, we exclude any hypertension-associated variants in the GRASP testing set that fall within 10 kb of any hypertension-associated variants found in the ARB training set. We then repeat the performance comparison experiment using the newly reduced testing set. The results are summarized in Additional file 1: Table S15. The predicted AUC values are shown in Additional file 1: Table S16. The average MCC values are shown in Additional file 1: Table S17.

From Additional file 1: Table S15, we see that despite slightly dampened performance, removing variants in the testing set that are close to variants in the training set does not change the fact that DIVAN significantly outperforms all the other competing methods that have been tested.

### Different size of benign set

Because there are much more benign variants than risk variants, it is an interesting question that how many benign variants should be included in the training set. In the CV described earlier, we choose the size of the benign variants to be ten times that of the risk variants. Here we investigate whether increasing the size of the benign set to 100 times of the risk set has any effect on the predictive performance. We calculate Pearson correlation coefficient between the two sets of CV-AUC values obtained from the two settings. We also summarize the mean and standard deviation of the CV-AUC values for each setting in Additional file 1: Table S18. The CV-AUC values are shown in Additional file 1: Table S19. The predicted AUC values are shown in Additional file 1: Tables S20 and S21. Our result suggests that, overall, increasing the size of the benign variant set when set up the training model does not change much in terms of the predictive performance in CV.

For the independent test, we also experiment with increasing the number of benign variants from ten times that of the risk variants to 100 times for each disease and check whether the different level of imbalance in the testing set has any effect on the prediction performance. The new predicted AUC values are shown in Additional file 1: Tables S22 and S23, where we could see that the AUC values remain stable on the 36 diseases in GRASP. The Pearson correlation coefficient between the two sets of 36 predicted AUC values is 0.999 (*p* value < 2.2e-16) when the number of benign variants is ten and 100 times of risk variants, respectively. Thus, we see that increasing the size of the benign variants has little effect on the predictive performance for the independent test, which suggests that the performance of DIVAN is not significantly affected with different level of risk/benign imbalance.

## Discussion

A key emphasis of DIVAN lies on disease specificity. We believe this can be achieved by using variants that are specific to that disease in the training set as opposed to including all variants that have shown associations with some diseases. Despite a small training set, we show that advanced statistical learning techniques can help us overcome this challenge and achieve better performance in identifying variants specific to that disease. Unlike existing approaches, DIVAN uses thousands of annotations from various public resources, including DNase-seq, FAIRE-seq, and TF/Histone ChIP-seq, across different cell types. The more annotations collected, the better the chance informative annotations will be discovered, resulting in a better chance of discriminating risk variants from benign ones. There is still room to improve DIVAN further. Other types of genomic and epigenomic features, including eQTL, DNA methylation, and pre-computed scores from GWAVA, CADD, and GenoCanyon, will also be added into DIVAN. Another important regulatory mechanism through which non-coding variants influence diseases is the disruption of splice junction and splicing enhancer [29]. The mutations effect on splice sites is similar to nonsense or missense mutations. A myriad of cases about splice site variants have been reported in the literature [30–34]. Because of this, we have decided to add a splicing-related feature, which is the distance to the splice sites (586,795 such sites can be found in Ensemble [35] release 70), into the next release of DIVAN. The same feature has been used in GWAVA.

Currently, to represent epigenomic features, existing methods use binary indicators showing whether a ChIP-seq peak overlaps with the variant. In DIVAN, we apply an alternative method in which continuous ChIP-seq read counts in the vicinity of the variant are used to represent epigenomic features. The advantage of using read count rather than peak presence as the feature lies on the former’s better sensitivity and ability to distinguish risk variants from benign ones with a limited number of epigenomic features and to detect significant differences in both enrichment and depletion (Additional file 2: Figure S9). Moreover, our analyses also show that using read count as the feature results in more informative features being included in the model, especially for features associated with TF binding.

One of the key findings from this study is that histone marks associated with repressive chromatin, in particular H3K9me3, turns out to be an important feature for risk variant identification. For most of the diseases, we find that this particular repressive mark is often among the top-ranked features, showing significant depletion around the risk variants compared to benign ones. Such a finding is consistent with what has been reported in the literature. In a recent study, Pickrell found that repressed chromatin is significantly depleted around SNPs associated with multiple phenotypes [27]. Chen et al. found that the binding regions of another repressive histone mark, H3K27me3, are significantly less likely to overlap with risk SNP blocks of prostate cancer [36]. Despite these findings, repressive chromatin marks do not play an important role in existing methods for risk variant annotation. For histone marks, almost all attention has been focused on the enrichment of active chromatin marks. For example, the three histone marks used in CADD and Eigen are H3K27ac, H3K4me1, and H3K4me3. A primary reason why only active chromatin marks are used is that it is easier to detect enrichment of a factor, but not depletion when peak is used as the feature. In contrast, using read around the variants as the feature, we are able to detect enrichment as well as depletion.

It is worth clarifying that the risk variants considered in this study are not necessarily “causal” variants since in most cases, no evidence beyond significant association *p* values derived from GWAS separates them from the millions of variants found throughout the genome. It would be interesting to test DIVAN using functionally validated variants as the training set. However, the number of such variants is very limited and insufficient for study on individual diseases today.

A potential application of DIVAN is personal genome sequencing interpretation. In the genome of an individual patient, it is expected that many novel, rare, and non-coding variants will be detected. Due to the sample size limitation, little information can be learned from GWASs for these rare variants. Alternatively, by looking at the surrounding regions of such variants and comparing to the genomic and epigenomic profiles of GWAS-associated risk variants represented by DIVAN, we can potentially gauge their impact on a particular disease. We have pre-computed DIVAN scores for every base in the human genome, which we believe will be a great resource for annotating rare and non-coding variants that would be identified in personal genome sequencing studies.

## Conclusion

In this work, we describe DIVAN, a feature selection-based ensemble learning framework for identifying disease-specific, non-coding risk variants. DIVAN performs favorably when compared to existing state-of-the-art methods, both supervised (CADD, GWAVA) and unsupervised (GenoCanyon, Eigen), for detecting disease-specific, non-coding risk variants. From a clinical perspective, it is of great practical and conceptual value to evaluate the impact of a variant on individual disease/phenotype. Because the number of disease-implicated variants is far fewer than the number of static genomic and epigenomic annotations for most diseases, to avoid potential over-fitting in the high-dimensional setting, we employ model selection to remove non-informative features. Besides feature selection, the ensemble method is adopted to improve the predictive performance due to the nature of the imbalance between risk variants and benign ones. This combination of feature selection and ensemble method makes DIVAN more powerful and robust.

Another major finding of the study is that the depletion of H3K9me3, a histone mark associated with repressed chromatin, is the most prominent hallmark around risk variants. Overall, histone marks contribute more informative features in risk variant identification than transcription factors and open chromatin in DIVAN. We believe the above findings have profound implications for understanding the mechanism behind the way non-coding variants make their impact on diseases/phenotypes via epigenetic modifications.

## Methods

### Software and data package availability

To maximize DIVAN’s utility, we pre-computed DIVAN score for every base of the human genome (hg19), and for each of the 45 diseases, using either the TSS-matched criterion or the region-matched criterion. DIVAN offers two options to query and retrieve these scores: by variant identifier (for known variants) or by genomic regions. For known variants, DIVAN allows the user to retrieve scores for all known variants found in the Ensembl variation database (release 70, including 49,999,357 variants), COSMIC database [37] (v78, including 3,153,949 variants by excluding variants on mitochondrial DNA and variants without genomic position), and 1000 Genome variants (Phase I, including 17,076,840 variants). For genomic regions, users can select either to retrieve scores from all known variants within the genomic regions or obtain the average base-level scores for each genomic region. Correspondingly, DIVAN provides R scripts for both options. The DIVAN software toolkit and the pre-computed scores are freely available at https://sites.google.com/site/emorydivan/ under the GNU General Public License v3.

### Data sources

#### Construction of disease-specific risk variants and benign variants

The risk variants chosen from ARB include 28,713 unique non-coding SNPs (12,159 intronic SNPs and 16,803 intergenic SNPs) spanning 555 diseases/phenotypes across 33 disease/phenotype classes. In the present study, to maintain enough risk variants in the training set, we chose 45 diseases/phenotypes spanning 12 disease/phenotype classes, with at least 50 disease-SNP associations. The 45 diseases/phenotypes with the numbers of risk variants are summarized in Additional file 1: Table S1.

To construct a set of benign variants for each disease/phenotype, we apply the same strategy used in GWAVA by sampling variants not reported to be disease-implicated and by requiring the distances between these benign variants and their nearest TSSs to have the same empirical distribution as those risk variants. All benign variants are sampled from the 1000 Genomes Project Phase I catalog (with minor allele frequency higher than 5%), excluding all variants found in the ARB. Similar to GWAVA, ten times more benign variants than risk variants are selected for each disease/phenotype.

#### Merge replicates

Most of the experiments in ENCODE and RMEC contain biological replicates. To simplify the analysis, we merge reads produced from replicated ChIP-seq experiments if both the factor (TF/Histone) and cell line are the same; reads from open chromatin experiments conducted on the same cell line are also merged. Since all ENCODE/REMC ChIP-seq experiments are performed with ChIP and matched input samples, we calculate the normalized read count by subtracting the number of input reads from the ChIP reads after adjusting the sequencing depth. For open chromatin experiments, DNase-seq and FAIRE-seq, we use the ChIP reads directly as there is no matching input sample. For pre-processed peak files of the same factor and the same cell line, overlapped peaks are merged by taking the union.

#### Annotation sources

##### Open chromatin

ENCODE conducts two types of sequencing experiments to profile genome-wide open chromatin regions: DNase-seq and FAIRE-seq. We include both in the feature collection for DIVAN. To be specific, for mapped read files, we collect 230 DNase-seq datasets (merged into 80 features) and 78 FAIRE–seq datasets (merged into 31 features) from ENCODE and 350 DNase-seq datasets (merged into 73 features) from REMC; for corresponding pre-processed peak files, we collect 100 DNase-peak files and 38 FAIRE-peak files (merged into 31 features) from ENCODE and 39 DNase-peak files from REMC.

##### Transcription factor binding sites (TFBS)

For mapped read files, we obtain 650 TF ChIP-seq datasets (merged into 292 features) from ENCODE/HAIB and 681 TF ChIP-seq datasets (merged into 279 features) from ENCODE/SYDH; for corresponding pre-processed peak files, we collect 638 TF-peak files (merged into 295 features) from ENCODE/HAIB and 321 TF-peak files (merged into 288 features) from ENCODE/SYDH.

##### RNA polymerase binding

For mapped read files, we collect 156 RNA polymerase binding ChIP-seq datasets (merged into 49 features); for corresponding pre-processed peak files, we collect 92 peak files (merged into 53 features) from ENCODE

##### Histone modification

We include histone ChIP-seq datasets from both ENCODE and REMC. For mapped read files, we collect 549 histone ChIP-seq datasets (merged into 267 features) from ENCODE and 1407 histone ChIP-seq datasets (merged into 735 features) from REMC; for corresponding pre-processed peak files, we collect 280 histone-peak files (merged into 270 features) from ENCODE and 979 histone ChIP-peak files from REMC.

##### Genomic features

Two types of static genomic features are included in DIVAN: repeated elements and conservation scores (genomic evolutionary rate profiling (GERP) element [38] and phastCon scores [39]). We consider all repeated elements collected in the UCSC Genome Browser, including LINE, low complexity, satellite, simple repeat, SINE, LTR, etc. Conservation annotations include GERP elements and phastCon score, which are known to influence the functional consequences of genetic variants, such as phylogenetic conservation and level of selective constraint. GREP elements are downloaded from the Sidow Lab (http://mendel.stanford.edu/SidowLab/downloads/gerp/) and further treated as a binary annotation for each variant investigated. The phastCon scores are calculated for variants of interest using Bioconductor package phastCons100way.UCSC.hg19.

#### Annotation segmentation

To simplify the computation, we first cut the whole genome into 200-bp bins and calculate the feature value, i.e., normalized mapped read count or the peak presence for each bin. Therefore, the result is a genome-wide annotation matrix with rows as 200-bp bins across the whole genome and columns as genomic and epigenomic features. With the pre-built genome-wide annotation matrix, we could easily retrieve feature values for each variant by simply determining which bin the variant falls into.

### Feature selection-based ensemble-learning framework

The workflow of DIVAN is illustrated in Fig. 1, which consists of four steps. The first step is to build the risk variant set and the benign variant set. All risk variants from the selected 45 diseases/phenotypes are retrieved from ASB. The benign variants are obtained from the 1000 Genomes Project. In the second step, variants in both sets are annotated by genomic and epigenomic sources, including GERP elements, phastCon scores, repeat elements, and genome-wide epigenomics profiling data collected from ENCODE and RMEC. The third step is selecting informative features. In the last step, an ensemble module, which is a collection of ensemble base learners, is developed to adjust the class imbalance between risk variant set and benign variant set. The base learner could be an arbitrary binary classifier. The default option is the decision tree. With the test variants annotated by the same source in the second step, the trained model would output the probability of being disease-implicated for each test variant.

#### Feature selection

We perform feature selection to avoid over-fitting since the number of features is far greater than the number of variants, which is a typical large *n*, small *p* problem.

As the confidence of a feature is measured by *p* values, we use different tests for different types of annotations to obtain the *p* values. For continuous features, e.g., number of reads, we use a two-sided t-test; for binary features, e.g., peak presence, we use Fisher’s exact test by constructing a two-by-two contingency table. Additional file 2: Figure S10A shows the distribution of t-statistics for all epigenomic features, with the heavy tail corresponding to the informative features. The distribution of corresponding *p* values is shown in Additional file 2: Figure S10B, while the *p* values obtained from Fisher’s exact test can be found in Additional file 2: Figure S10C. By comparing the distribution of *p* values for the two tests, we find that *p* values from Fisher’s exact test are right-skewed compared to the left-skewed t-test *p* values. This observation indicates fewer informative features would be selected if peak is used as the feature.

After obtaining the *p* values for all features, we use cross-validation to define the *p* value threshold in the feature selection step and features with a *p* value below the threshold are considered as informative features. To be specific, we set a sequence of possible *p* value thresholds. For each threshold, the mean of the predicted AUC values is calculated using fivefold cross-validation on the training set and the *p* value threshold is chosen as the one with the largest predicted AUC value. In fact, the selected *p* value threshold could be considered as a tuning parameter.

#### Choosing the appropriate base learner

Three classifier engines have been evaluated as a base learner in the ensemble module of DIVAN: decision tree, support vector machine (SVM), and Lasso. For SVM, we use non-linear classifiers with radial kernel. For Lasso, we perform fivefold cross-validation to choose the best tuning parameter for **L**_1_ penalty. Additional file 2: Figure S11A and B shows that even if decision tree, Lasso, and SVM have comparable AUC values, decision tree shows a better precision–recall curve. Thus, decision tree is chosen as the default base learner for the ensemble module.

#### Ensemble method for class imbalance adjustment

The number of benign variants far exceeds the number of disease-associated variants, which makes the task of discriminating disease-specific risk variants from benign ones an inherent imbalanced two-class classification problem. A single binary classifier usually has poor predictive performance without adjusting the class imbalance. To build a balanced classifier without downsizing or duplicating the training set, we adopt an ensemble learning approach, which not only keeps all variants in the training set but also overcomes the class imbalance issue. We formularize the ensemble method as below.

We denote the benign set as *N*, the risk variant set as *P*, and the number of base learners as *C*. Specifically, we create two balanced classes by sampling the same number of variants *N*_*i*_ with replacement from the benign set as the number of variants *C* in the risk variant set to form one training set *N*_*i*_ ∪ *P* for base learner *C*_*i*_. The choice of number of base learner *c* would be large enough to ensure the unions of all *N*_*i*_ (*N*_1_ ∪ *N*_2_, …, ∪ *N*_*C*_ ) could cover most of *N*. The default *c* is set to be twice the number of benign variants in *N* over risk variants in *P*. We further denote the annotation matrix for variants in *N*_*i*_ ∪ *P* as *X*_*train*_^*i*^ and the labeled *N*_*i*_ ∪ *P* as *Y*_*train*_^*i*^, the trained ensemble module is formulated as a function of training sets, which is ***f***(***X***, ***Y***) = *c*(*f*(*X*_*train*_^1^, *Y*_*train*_^1^), *f*(*X*_*train*_^2^, *Y*_*train*_^2^), … (*X*_*train*_^*c*^, *Y*_*train*_^*c*^)). With a given variant with annotation matrix *X*_*test*_, the probability of the given variant being disease-implicated is the average of all predictive probabilities of base learners,

$$ \mathrm{E}\left({\mathrm{Y}}_{\mathrm{test}}=1\Big|{\mathrm{X}}_{\mathrm{test}},\mathrm{f}\left(\mathrm{X},\mathrm{Y}\right)\right)=\frac{1}{\mathrm{C}}{\displaystyle \sum_{\mathrm{i}=1}^{\mathrm{C}}}\mathrm{E}\left({\mathrm{Y}}_{\mathrm{test}}=1\Big|{\mathrm{X}}_{\mathrm{test}},\mathrm{f}\left({\mathrm{X}}_{\mathrm{train}}^{\mathrm{i}},{\mathrm{Y}}_{\mathrm{train}}^{\mathrm{i}}\right)\right) $$

#### Competing methods

We compare DIVAN with four existing risk variant annotation and prioritization methods: GWAVA, CADD, Eigen, and GenoCanyon.

##### Supervised methods: GWAVA and CADD

CADD is a SVM-based supervised learning method. It maintains a database of pre-computed C-scores for 1000 Genomes variants and base levels for the whole human genome. GWAVA is a random forest-based supervised learning method. It maintains a database containing three sets of pre-computed scores for 1000 Genomes variants (minor allele frequency > 1%) based on different choices of benign variants (TSS, unmatched, and region).

##### Unsupervised methods: GenoCanyon and Eigen

GenoCanyon is an unsupervised learning method, which is a two-component mixture model. It maintains a database of base-level pre-computed scores across the whole human genome. Eigen, another unsupervised learning method, is also a two-component mixture model; however, it considers feature correlation. Eigen maintains a database containing two sets of pre-computed scores for 1000 Genomes variants. One is an Eigen score and another is a variation of Eigen score, EigenPC score.

For each of the above methods, we download and retrieve the pre-computed scores for the risk and benign variants. The scores are designed such that the higher the score, the better chance the variant is disease-associated. For GWAVA, we only report the set of scores with the best performance. For Eigen, we include both Eigen and EigenPC scores in the method comparison.

## Additional files

Additional file 1: Supplementary **Tables S1–S23.** Supplementary tables with legends in the first sheet called “Supplementary Tables Legends.” (XLSX 93 kb)
Additional file 2: Supplementary **Figures S1–S11.** Supplementary figures with legends in the pages (1–4). (PDF 2596 kb)
