# Supplementary material for: DIVAN: accurate identification of non-coding disease-specific risk variants using multi-omics profiles
Source: Genome Biol. 2016 Dec 6;17:252. doi: 10.1186/s13059-016-1112-z (PMC5139035; doi:10.1186/s13059-016-1112-z)
Supplement: Additional file 2: — Supplementary Figures S1–S11. Supplementary figures with legends in the pages (1–4). (PDF 2596 kb) [file 13059_2016_1112_MOESM2_ESM.pdf]

DIVAN: accurate identification of non-coding disease-specific risk variants using multi-omics profiles

All Supplemental Figures

Figure S1: Precision-recall curves of five-fold cross-validation on four diseases: carotid artery disease, macular degeneration, ulcerative colitis, and multiple sclerosis.

Figure S2: ROC curves of five-fold cross-validation for 41 diseases.

Figure S3: Cross-disease prediction shows the necessity for disease specificity of variant prioritization by using four diseases from four different disease classes: carotid artery disease (cardiovascular disease), Alzheimer's disease (mental disease), multiple sclerosis (immune disease), and macular degeneration (eye disease). (A) ROC curves showing that disease-specific prediction outperforms cross-disease prediction. **(B)** Heatmap showing that disease-specific prediction outperforms cross-disease prediction.

Figure S4: Precision-recall curves showing the effectiveness of the feature selection and ensemble method by comparing feature selection and ensemble combined, feature selection only, ensemble only, and the

baseline case: neither feature selection nor ensemble.

Figure S5: Contribution of three different feature groups, TF binding/histone modification/open chromatin, on prediction in four diseases: carotid artery disease, macular degeneration, ulcerative colitis, and multiple sclerosis. (A) ROC curves showing the predictive performance using each of the three feature groups of epigenomic features in the predictive model: TF binding, histone modification, and open chromatin, with read as the continuous feature for the four diseases. **(B)** ROC curves showing the predictive performance using each of the three feature groups of epigenomic features in the predictive model, TF binding, histone modification, and open chromatin, with peak as the binary feature for the four diseases.

Figure S6: ROC curves showing predictive performance of disease-class variant prioritization for immune diseases, including rheumatoid arthritis, asthma, type 1 diabetes mellitus, systemic lupus erythematosus, and multiple sclerosis. The ROC curves are generated by using a "leave-one-disease-out" approach; that is, the predictive model is built using variants of all other diseases within the disease class, tested on the variants of the disease being left out.

Figure S7: Bar chart showing the number of informative features for three feature categories (TF binding/histone modification/open chromatin) for 45 diseases when using peak as the feature.

Figure S8: Distribution of distances between non-coding SNPs associated with the 45 diseases in ARB to their nearest TSS (ignoring stand).

Figure S9: Distribution of enrichment proportions of H3K9me3 features and open chromatin features when using peak and read as the feature. The enrichment proportion is defined as the ratio between average read counts of each H3K9me3/open chromatin feature on risk variants and benign variants respectively.

Figure S10: Distributions of test statistics and p-values calculated between risk variants and benign variants for four diseases: carotid artery disease, macular degeneration, ulcerative colitis, and multiple sclerosis. (A) Distribution of t-test statistics using read as the feature for the four diseases. **(B)** Distribution of p-values of t-test for the four diseases. **(C)** Distribution of p-values of Fisher's exact test using peak as the feature for the four diseases.

Figure S11: ROC curves and precision-recall curves of five-fold

cross-validation using three base learners, decision tree, SVM, and Lasso, for four diseases: carotid artery disease, macular degeneration, ulcerative colitis, and multiple sclerosis. (A) ROC curves of five-fold cross-validation for three base learners: decision tree, SVM, and Lasso on the four diseases **(B)** Precision-recall curves of five-fold cross-validation for three base learners, decision tree, SVM, and Lasso, on the four diseases.

Figure S1

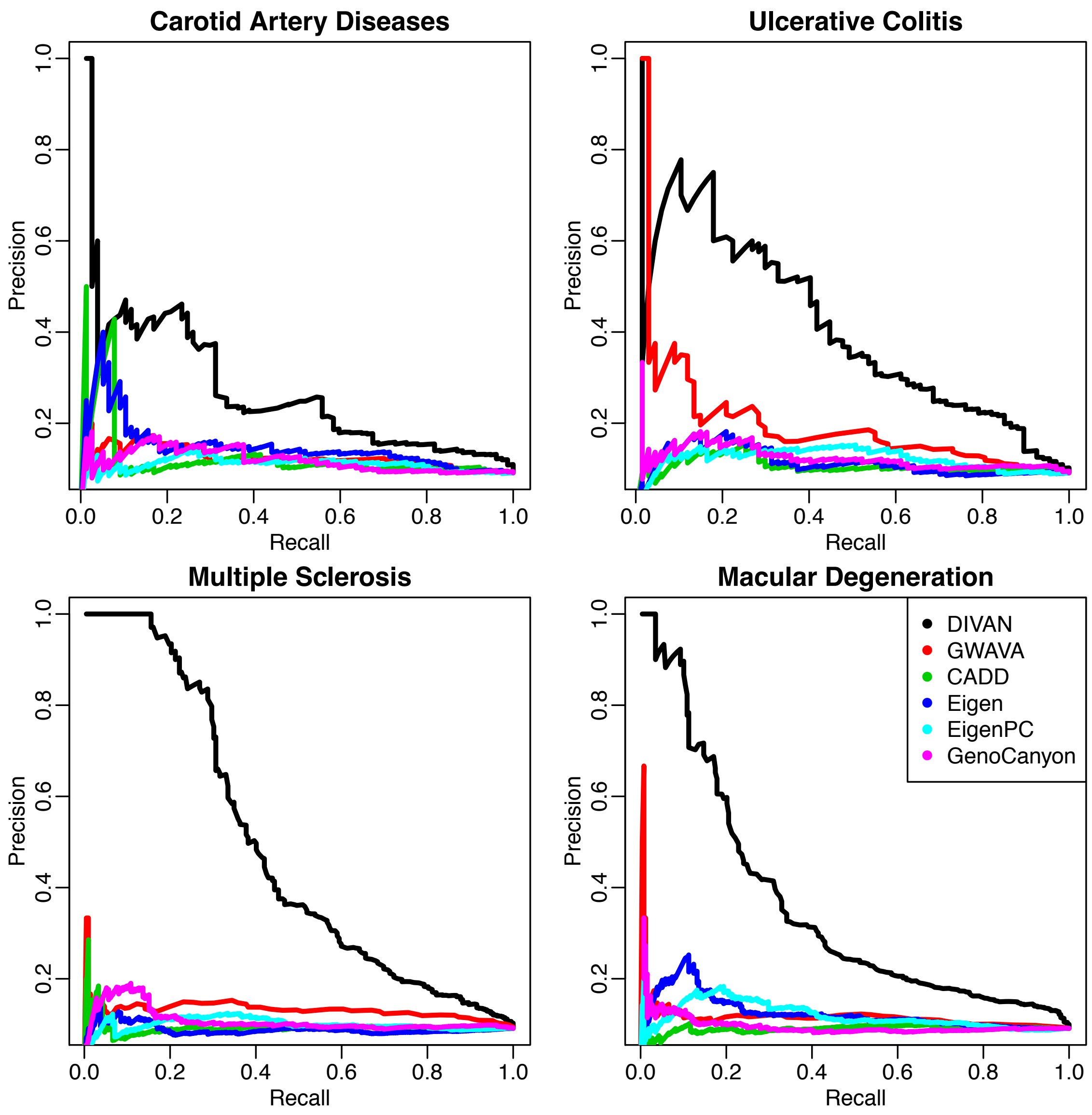

Carotid Artery Diseases
Ulcerative Colitis
Multiple Sclerosis
Macular Degeneration
Precision
Recall
0.0
0.2
0.4
0.6
0.8
1.0
DIVAN
GWAVA
CADD
Eigen
EigenPC
GenoCanyon

Figure S2

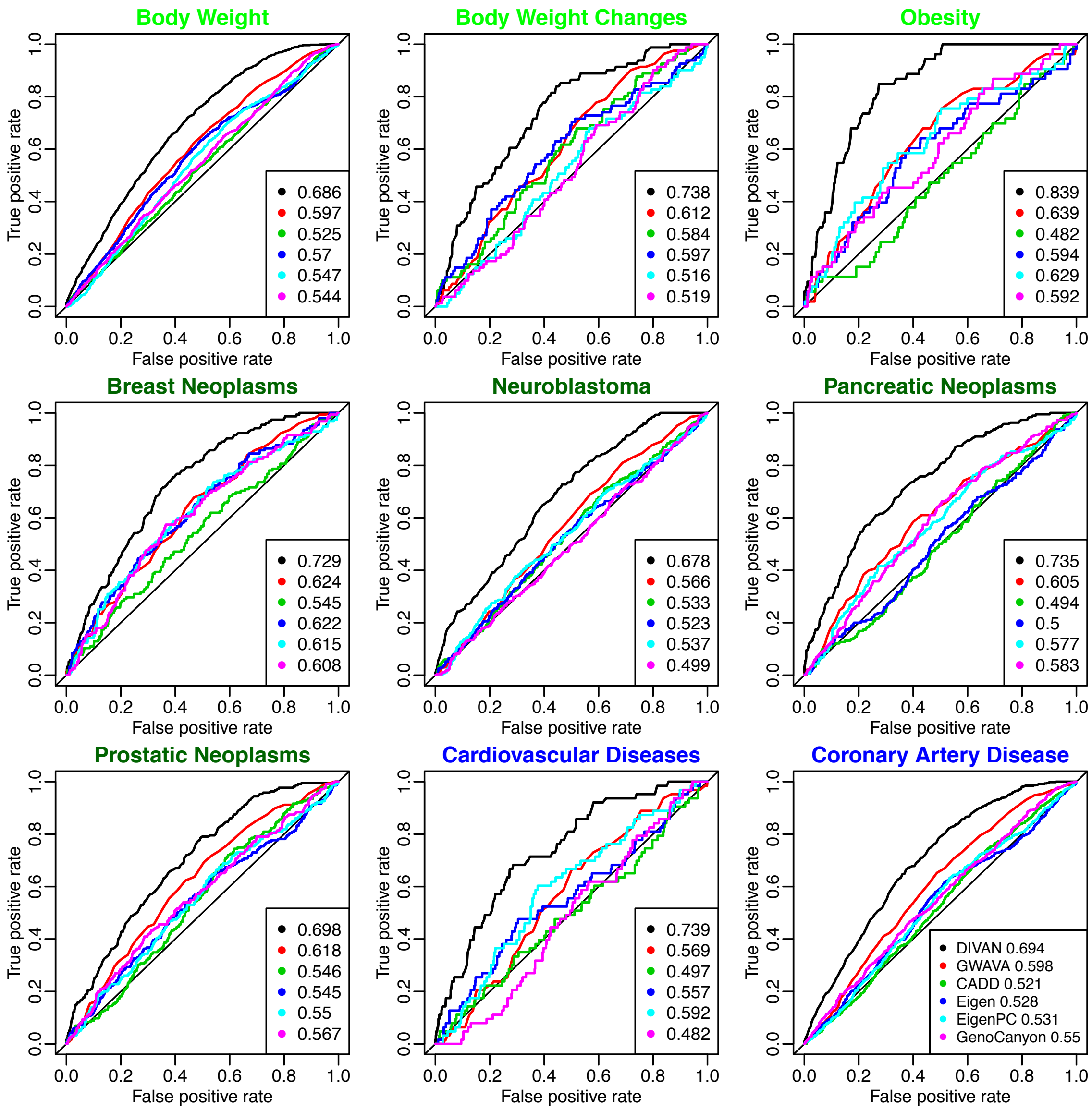

Body Weight
0.686
0.597
0.525
0.57
0.547
0.544
Body Weight Changes
0.738
0.612
0.584
0.597
0.516
0.519
Obesity
0.839
0.639
0.482
0.594
0.629
0.592
Breast Neoplasms
0.729
0.624
0.545
0.622
0.615
0.608
Neuroblastoma
0.678
0.566
0.533
0.523
0.537
0.499
Pancreatic Neoplasms
0.735
0.605
0.494
0.5
0.577
0.583
Prostatic Neoplasms
0.698
0.618
0.546
0.545
0.55
0.567
Cardiovascular Diseases
0.739
0.569
0.497
0.557
0.592
0.482
Coronary Artery Disease
DIVAN 0.694
GWAVA 0.598
CADD 0.521
Eigen 0.528
EigenPC 0.531
GenoCanyon 0.55
True positive rate
False positive rate

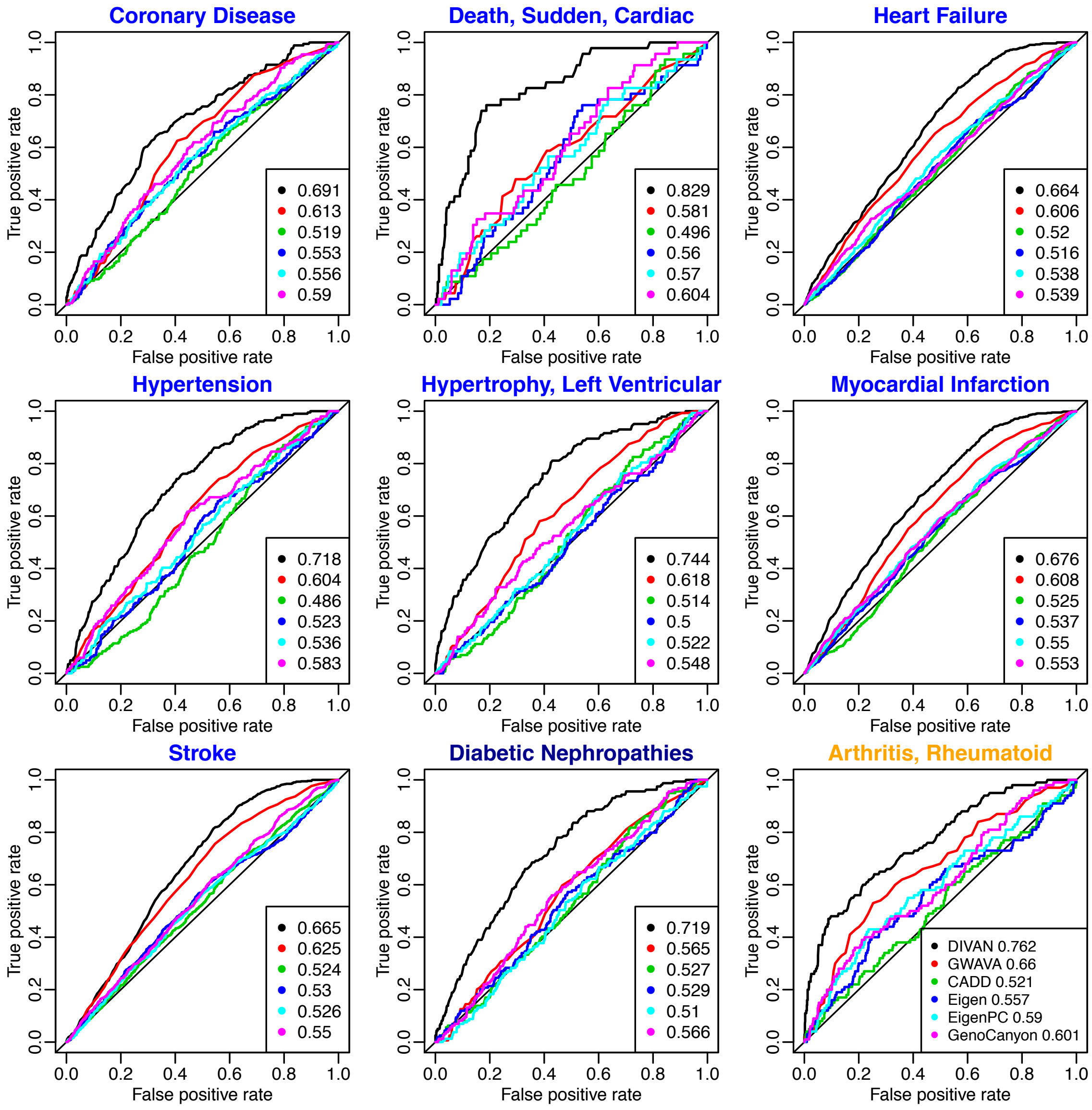
Coronary Disease
0.691
0.613
0.519
0.553
0.556
0.59
Death, Sudden, Cardiac
0.829
0.581
0.496
0.56
0.57
0.604
Heart Failure
0.664
0.606
0.52
0.516
0.538
0.539
Hypertension
0.718
0.604
0.486
0.523
0.536
0.583
Hypertrophy, Left Ventricular
0.744
0.618
0.514
0.5
0.522
0.548
Myocardial Infarction
0.676
0.608
0.525
0.537
0.55
0.553
Stroke
0.665
0.625
0.524
0.53
0.526
0.55
Diabetic Nephropathies
0.719
0.565
0.527
0.529
0.51
0.566
Arthritis, Rheumatoid
DIVAN 0.762
GWAVA 0.66
CADD 0.521
Eigen 0.557
EigenPC 0.59
GenoCanyon 0.601
True positive rate
False positive rate
0.0
0.2
0.4
0.6
0.8
1.0

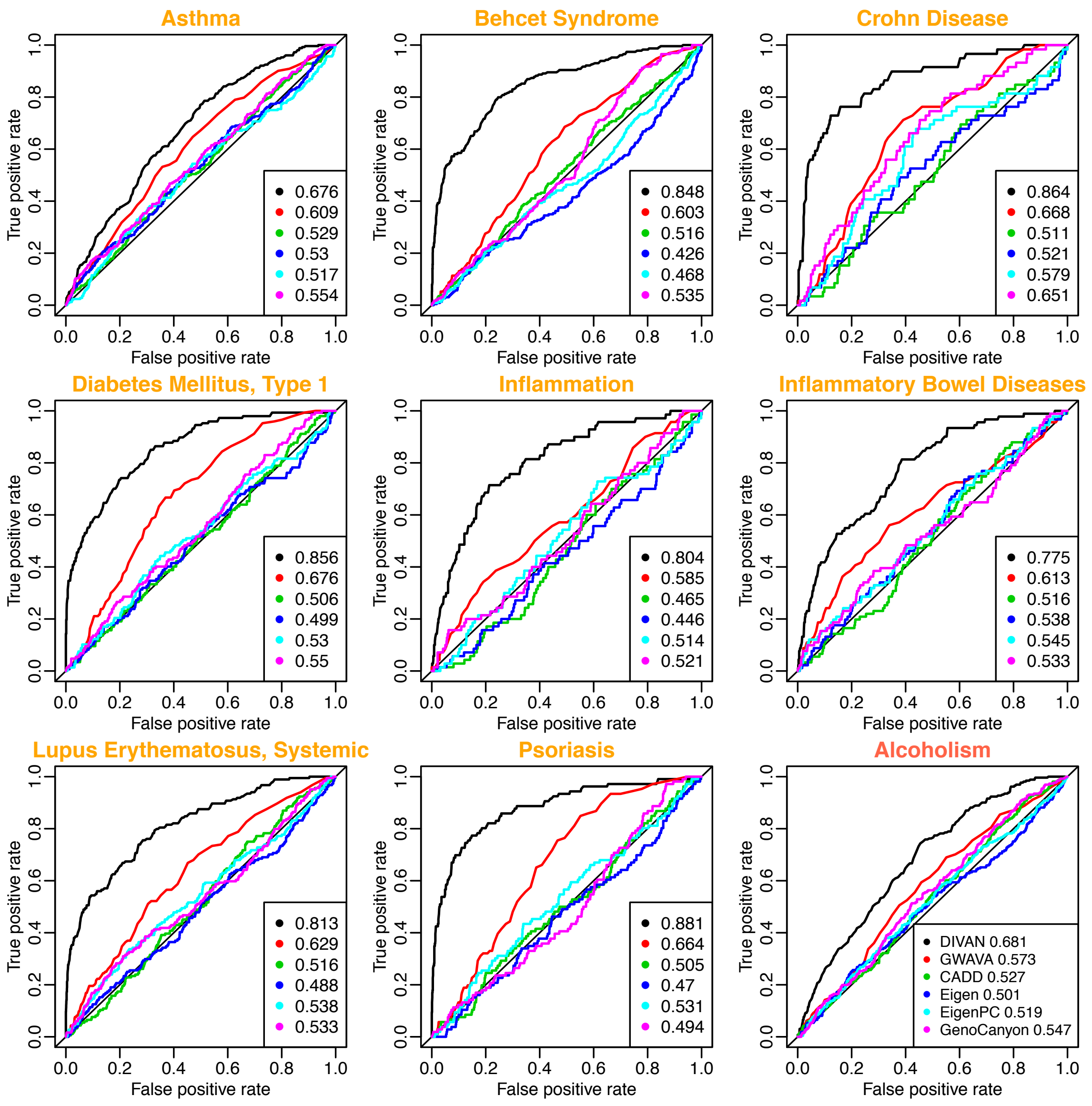

Asthma
0.676
0.609
0.529
0.53
0.517
0.554
Behcet Syndrome
0.848
0.603
0.516
0.426
0.468
0.535
Crohn Disease
0.864
0.668
0.511
0.521
0.579
0.651
Diabetes Mellitus, Type 1
0.856
0.676
0.506
0.499
0.53
0.55
Inflammation
0.804
0.585
0.465
0.446
0.514
0.521
Inflammatory Bowel Diseases
0.775
0.613
0.516
0.538
0.545
0.533
Lupus Erythematosus, Systemic
0.813
0.629
0.516
0.488
0.538
0.533
Psoriasis
0.881
0.664
0.505
0.47
0.531
0.494
Alcoholism
DIVAN 0.681
GWAVA 0.573
CADD 0.527
Eigen 0.501
EigenPC 0.519
GenoCanyon 0.547
True positive rate
False positive rate

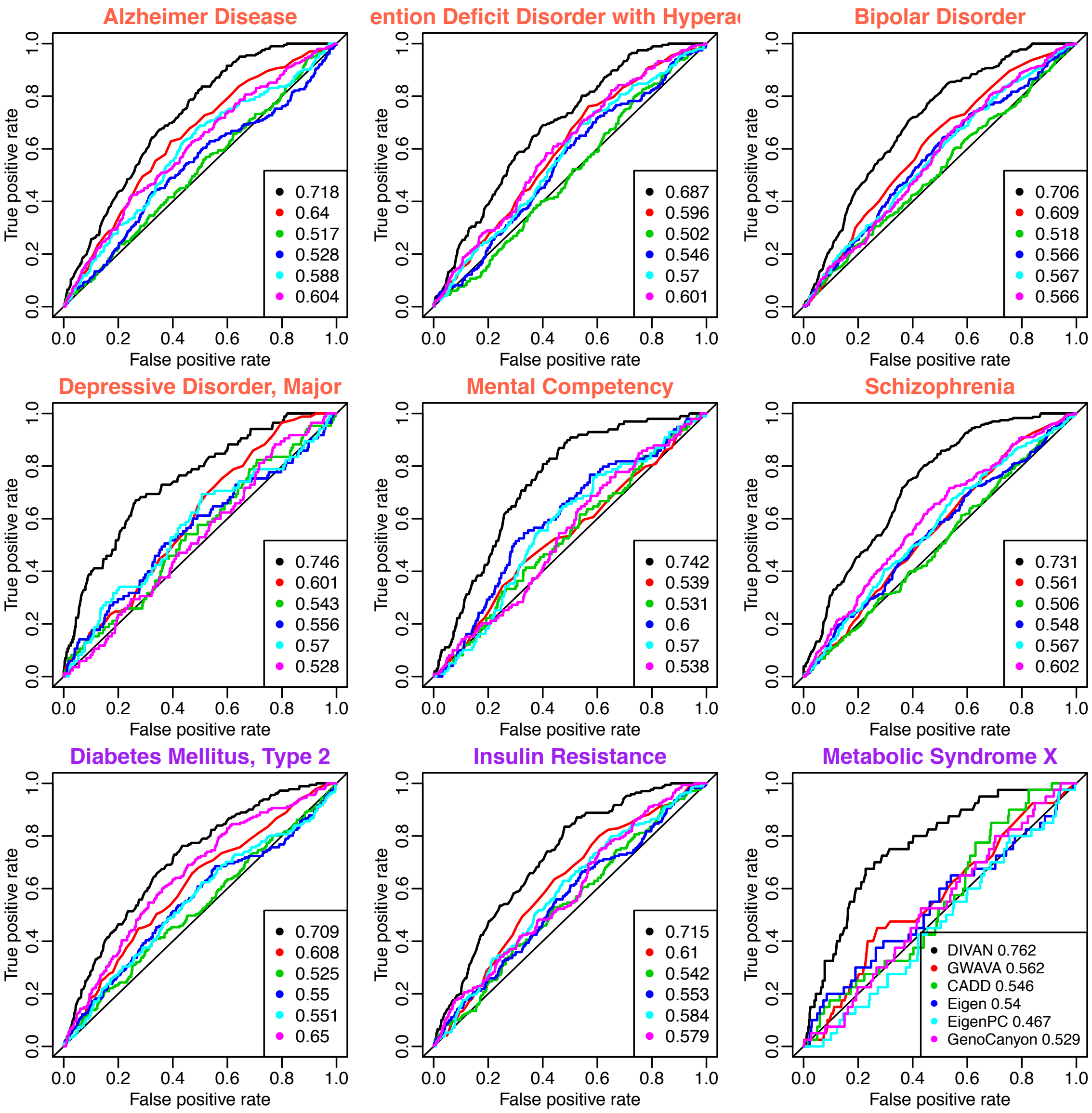
Alzheimer Disease
0.718
0.64
0.517
0.528
0.588
0.604
ention Deficit Disorder with Hyperac
0.687
0.596
0.502
0.546
0.57
0.601
Bipolar Disorder
0.706
0.609
0.518
0.566
0.567
0.566
Depressive Disorder, Major
0.746
0.601
0.543
0.556
0.57
0.528
Mental Competency
0.742
0.539
0.531
0.6
0.57
0.538
Schizophrenia
0.731
0.561
0.506
0.548
0.567
0.602
Diabetes Mellitus, Type 2
0.709
0.608
0.525
0.55
0.551
0.65
Insulin Resistance
0.715
0.61
0.542
0.553
0.584
0.579
Metabolic Syndrome X
DIVAN 0.762
GWAVA 0.562
CADD 0.546
Eigen 0.54
EigenPC 0.467
GenoCanyon 0.529
True positive rate
False positive rate
0.0
0.2
0.4
0.6
0.8
1.0

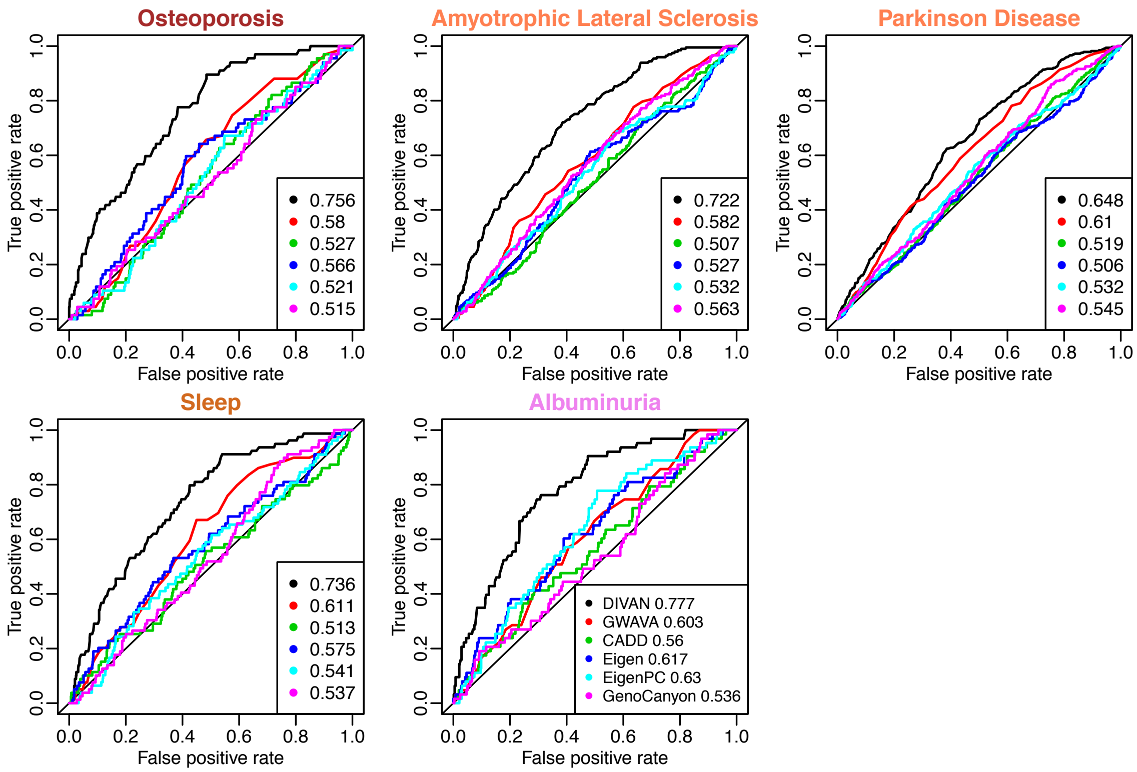

Osteoporosis
0.756
0.58
0.527
0.566
0.521
0.515
Amyotrophic Lateral Sclerosis
0.722
0.582
0.507
0.527
0.532
0.563
Parkinson Disease
0.648
0.61
0.519
0.506
0.532
0.545
Sleep
0.736
0.611
0.513
0.575
0.541
0.537
Albuminuria
DIVAN 0.777
GWAVA 0.603
CADD 0.56
Eigen 0.617
EigenPC 0.63
GenoCanyon 0.536
True positive rate
False positive rate
0.0
0.2
0.4
0.6
0.8
1.0

Figure S3-A

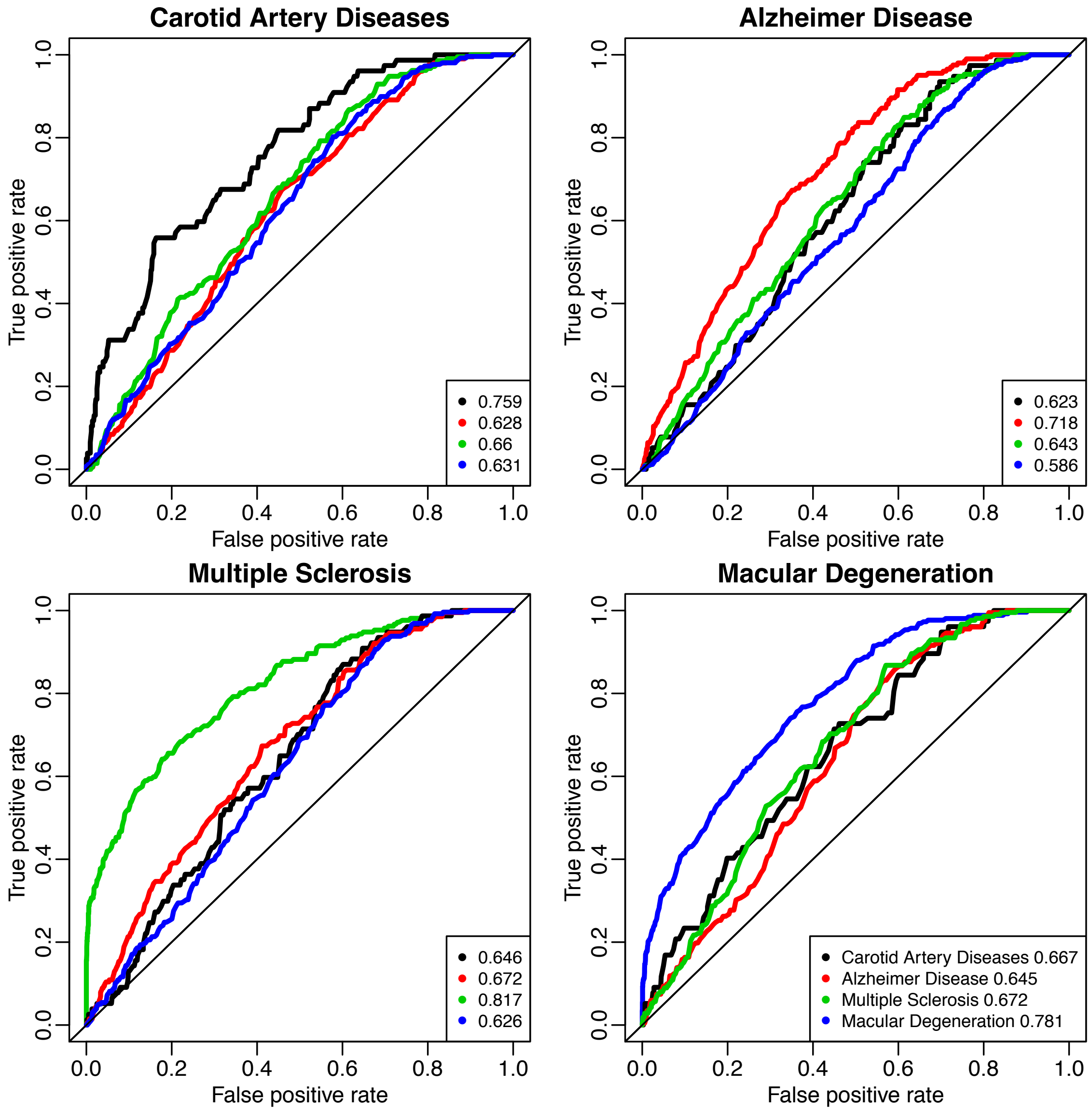

Carotid Artery Diseases
Alzheimer Disease
Multiple Sclerosis
Macular Degeneration
True positive rate
False positive rate
0.0
0.2
0.4
0.6
0.8
1.0
0.759
0.628
0.66
0.631
0.623
0.718
0.643
0.586
0.646
0.672
0.817
0.626
Carotid Artery Diseases 0.667
Alzheimer Disease 0.645
Multiple Sclerosis 0.672
Macular Degeneration 0.781

Figure S3-B

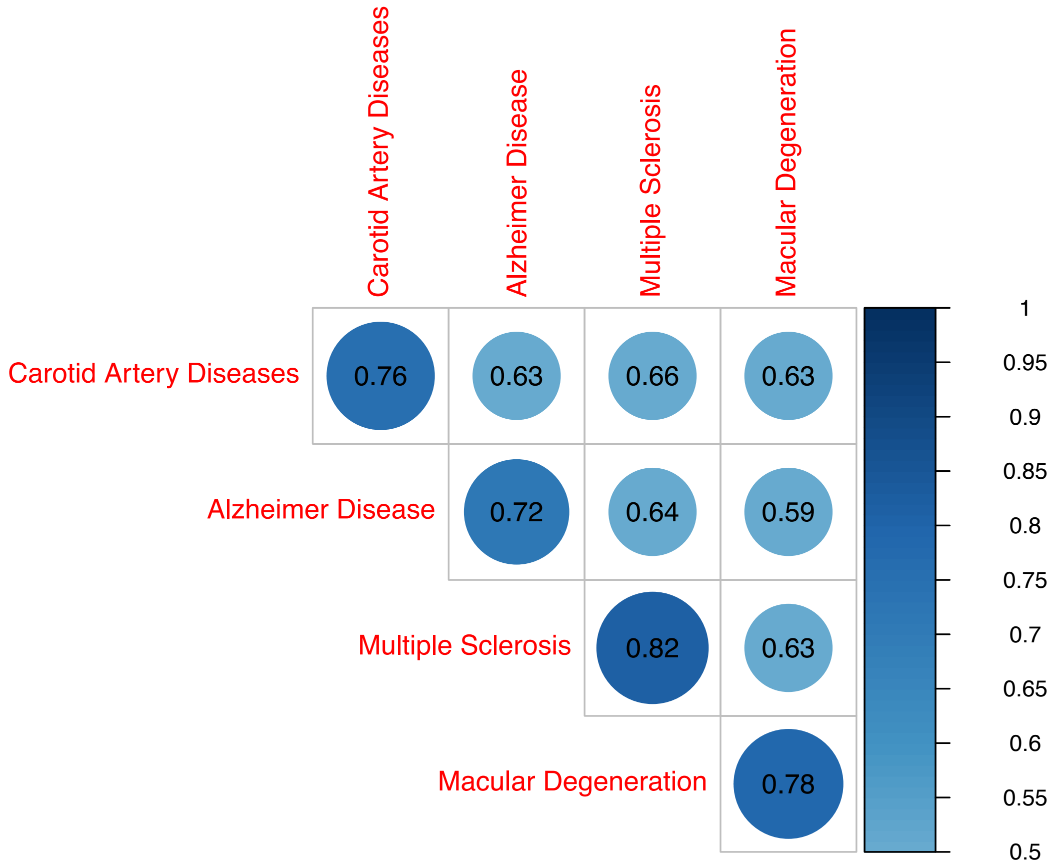

Carotid Artery Diseases
Alzheimer Disease
Multiple Sclerosis
Macular Degeneration
Carotid Artery Diseases
0.76
0.63
0.66
0.63
Alzheimer Disease
0.72
0.64
0.59
Multiple Sclerosis
0.82
0.63
Macular Degeneration
0.78
1
0.95
0.9
0.85
0.8
0.75
0.7
0.65
0.6
0.55
0.5

Figure S4

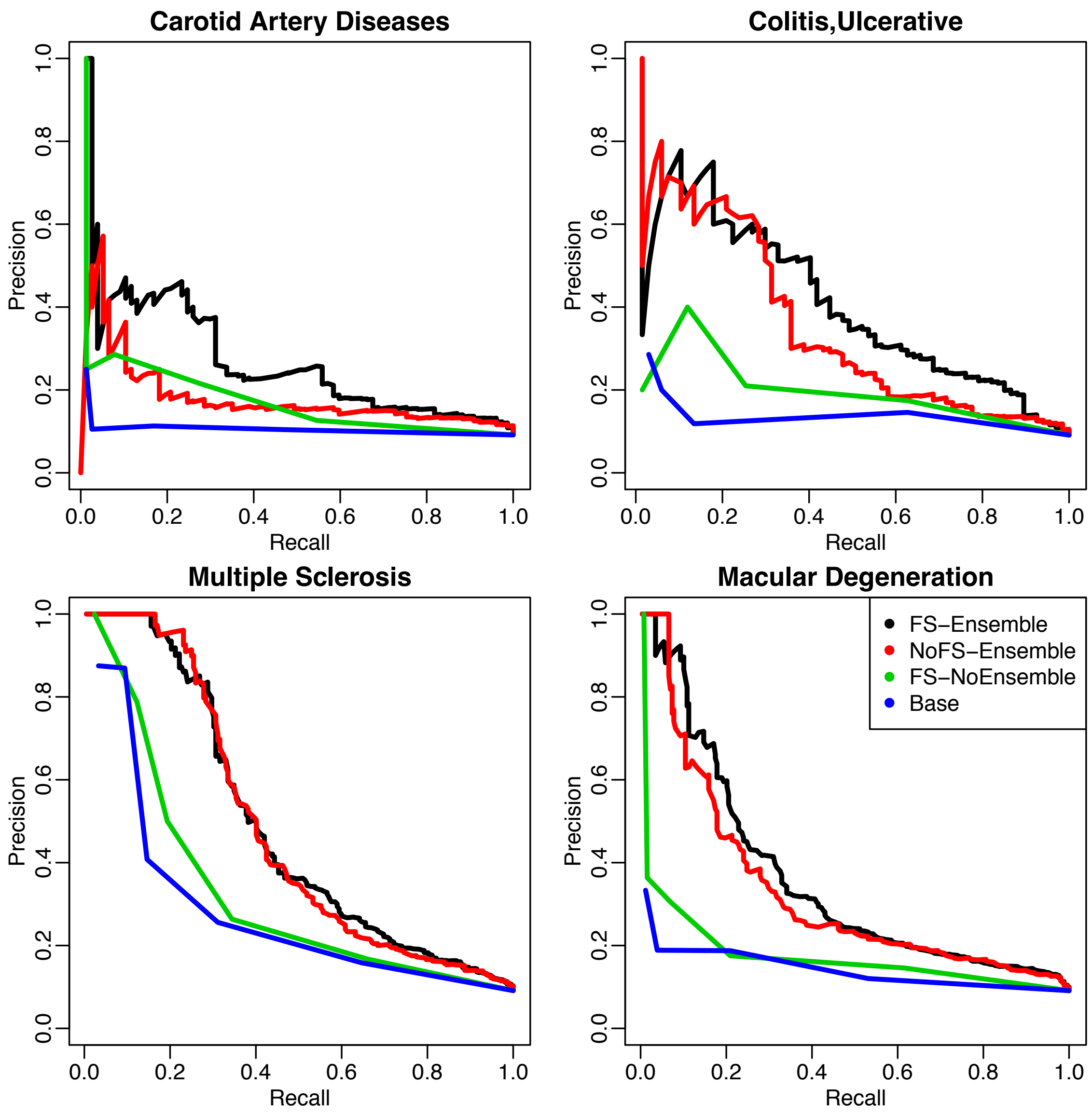

Carotid Artery Diseases
Colitis,Ulcerative
Multiple Sclerosis
Macular Degeneration
Precision
Recall
0.0
0.2
0.4
0.6
0.8
1.0
FS−Ensemble
NoFS−Ensemble
FS−NoEnsemble
Base

Figure S5-A

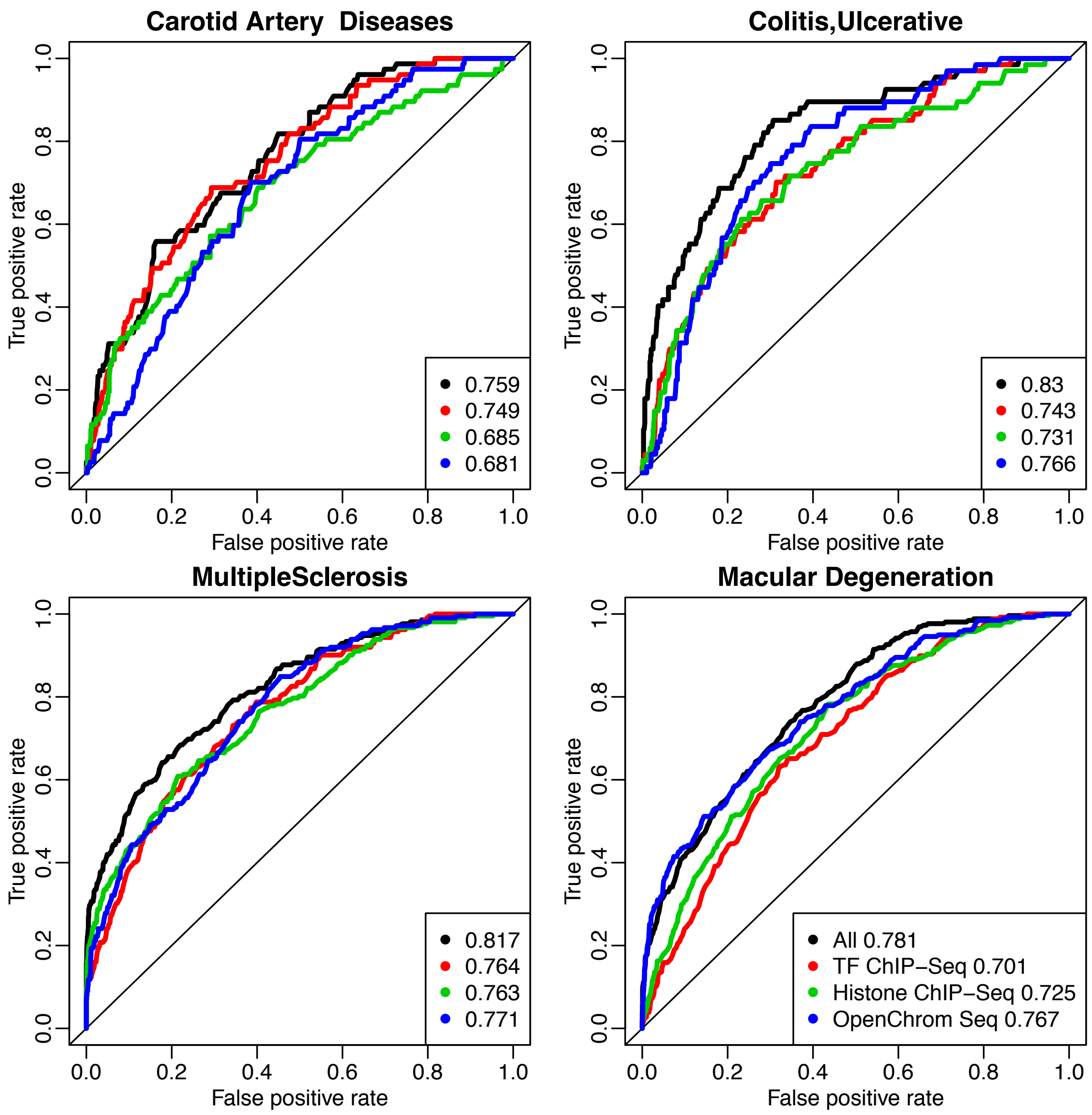
Carotid Artery Diseases
True positive rate
False positive rate
0.759
0.749
0.685
0.681
Colitis,Ulcerative
0.83
0.743
0.731
0.766
MultipleSclerosis
0.817
0.764
0.763
0.771
Macular Degeneration
All 0.781
TF ChIP−Seq 0.701
Histone ChIP−Seq 0.725
OpenChrom Seq 0.767

Figure S5-B

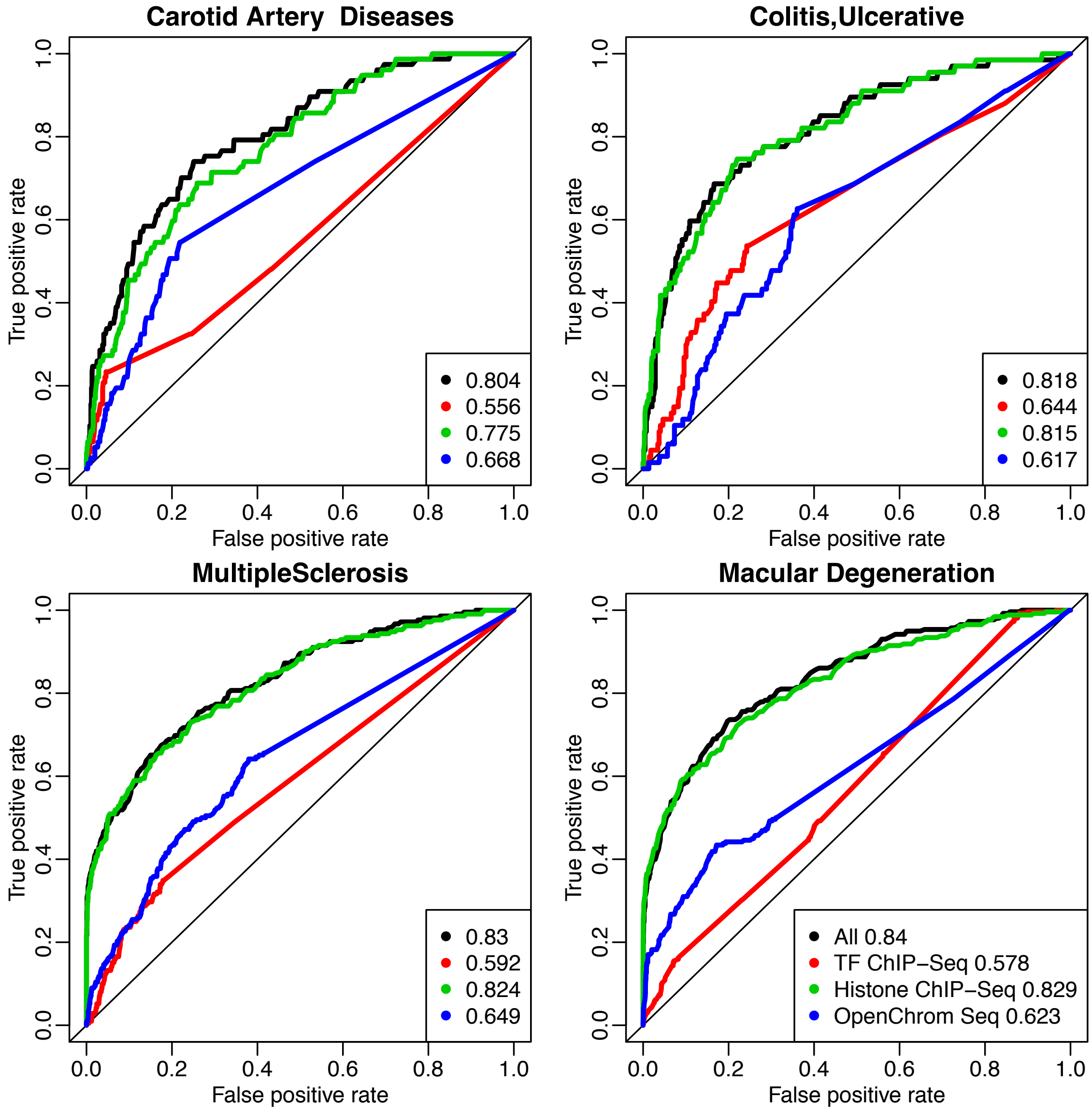
Carotid Artery Diseases
True positive rate
False positive rate
0.0
0.2
0.4
0.6
0.8
1.0
0.804
0.556
0.775
0.668
Colitis,Ulcerative
0.818
0.644
0.815
0.617
MultipleSclerosis
0.83
0.592
0.824
0.649
Macular Degeneration
All 0.84
TF ChIP−Seq 0.578
Histone ChIP−Seq 0.829
OpenChrom Seq 0.623

Figure S6

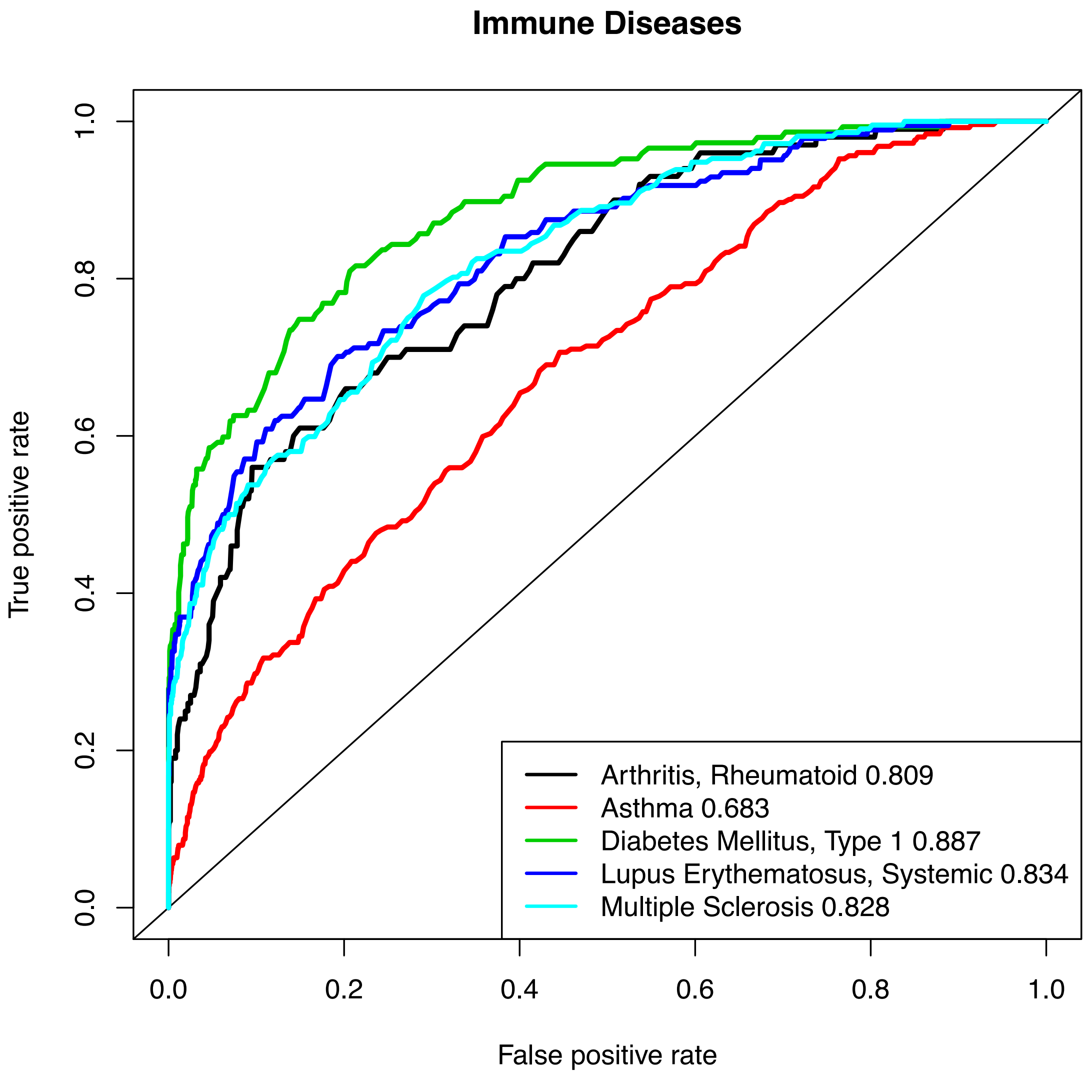
Immune Diseases
True positive rate
0.0
0.2
0.4
0.6
0.8
1.0
False positive rate
0.0
0.2
0.4
0.6
0.8
1.0
Arthritis, Rheumatoid 0.809
Asthma 0.683
Diabetes Mellitus, Type 1 0.887
Lupus Erythematosus, Systemic 0.834
Multiple Sclerosis 0.828

Figure S7

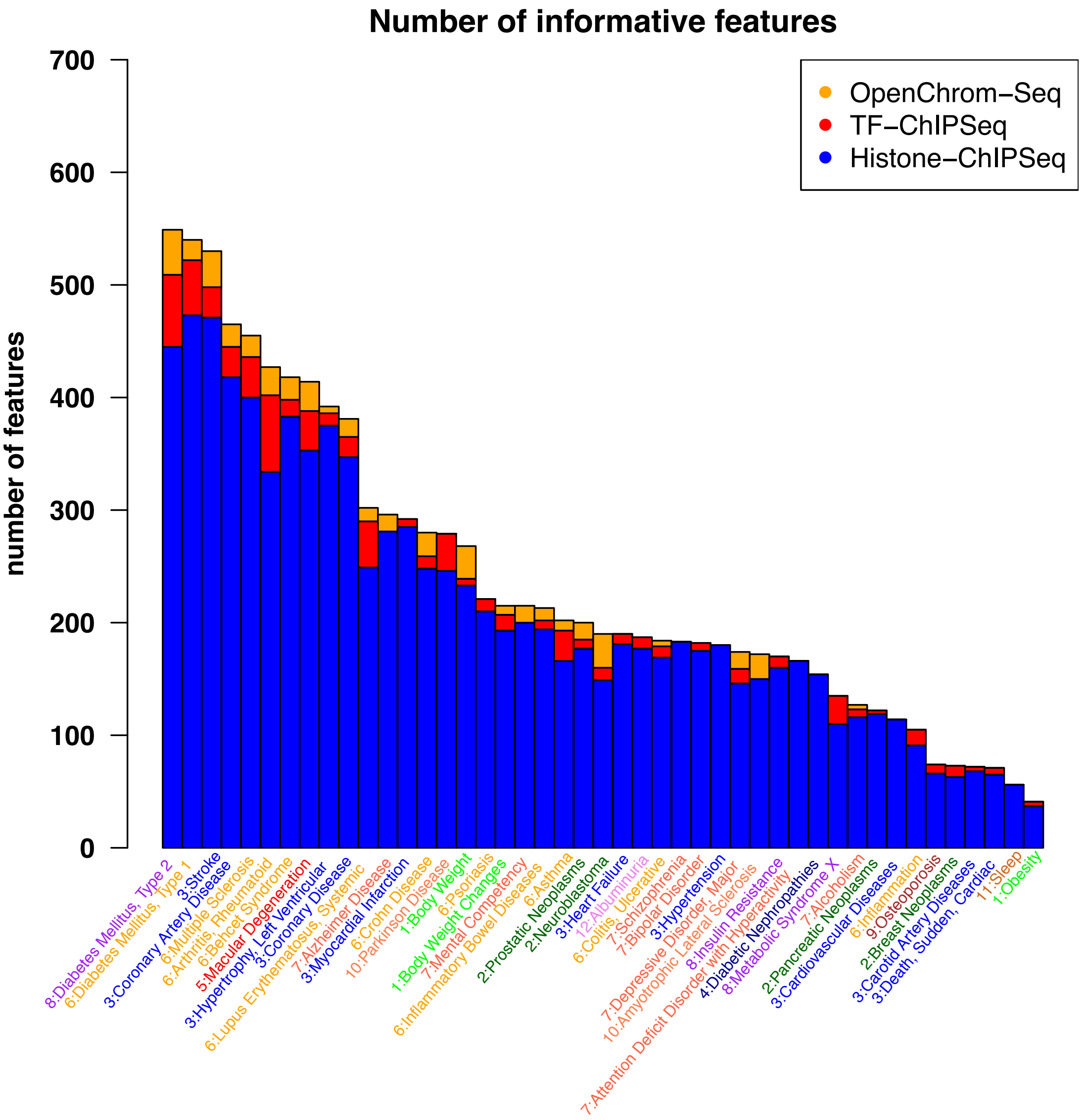

Number of informative features
number of features
0
100
200
300
400
500
600
700
OpenChrom−Seq
TF−ChIPSeq
Histone−ChIPSeq
8:Diabetes Mellitus, Type 2
6:Diabetes Mellitus, Type 1
3:Stroke
3:Coronary Artery Disease
6:Multiple Sclerosis
6:Arthritis, Rheumatoid
6:Behcet Syndrome
5:Macular Degeneration
3:Hypertrophy, Left Ventricular
3:Coronary Disease
6:Lupus Erythematosus, Systemic
7:Alzheimer Disease
3:Myocardial Infarction
6:Crohn Disease
10:Parkinson Disease
1:Body Weight
6:Psoriasis
1:Body Weight Changes
7:Mental Competency
6:Inflammatory Bowel Diseases
6:Asthma
2:Prostatic Neoplasms
2:Neuroblastoma
3:Heart Failure
12:Albuminuria
6:Colitis, Ulcerative
7:Schizophrenia
7:Bipolar Disorder
3:Hypertension
7:Depressive Disorder, Major
10:Amyotrophic Lateral Sclerosis
8:Insulin Resistance
7:Attention Deficit Disorder with Hyperactivity
4:Diabetic Nephropathies
8:Metabolic Syndrome X
7:Alcoholism
2:Pancreatic Neoplasms
3:Cardiovascular Diseases
6:Inflammation
9:Osteoporosis
2:Breast Neoplasms
3:Carotid Artery Diseases
3:Death, Sudden, Cardiac
11:Sleep
1:Obesity

Figure S8

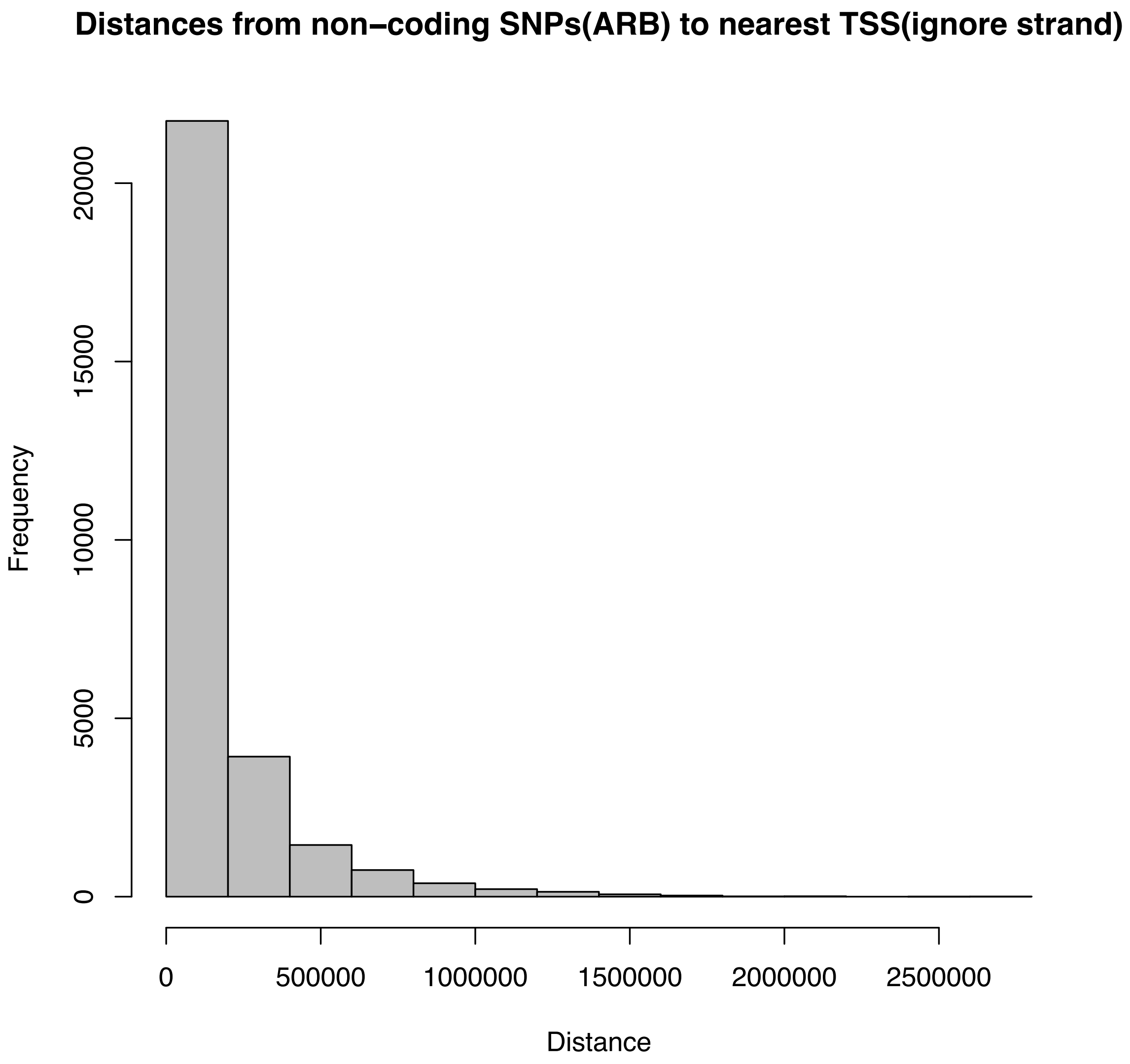

Distances from non−coding SNPs(ARB) to nearest TSS(ignore strand)
Frequency
0
5000
10000
15000
20000
0
500000
1000000
1500000
2000000
2500000
Distance

Figure S9

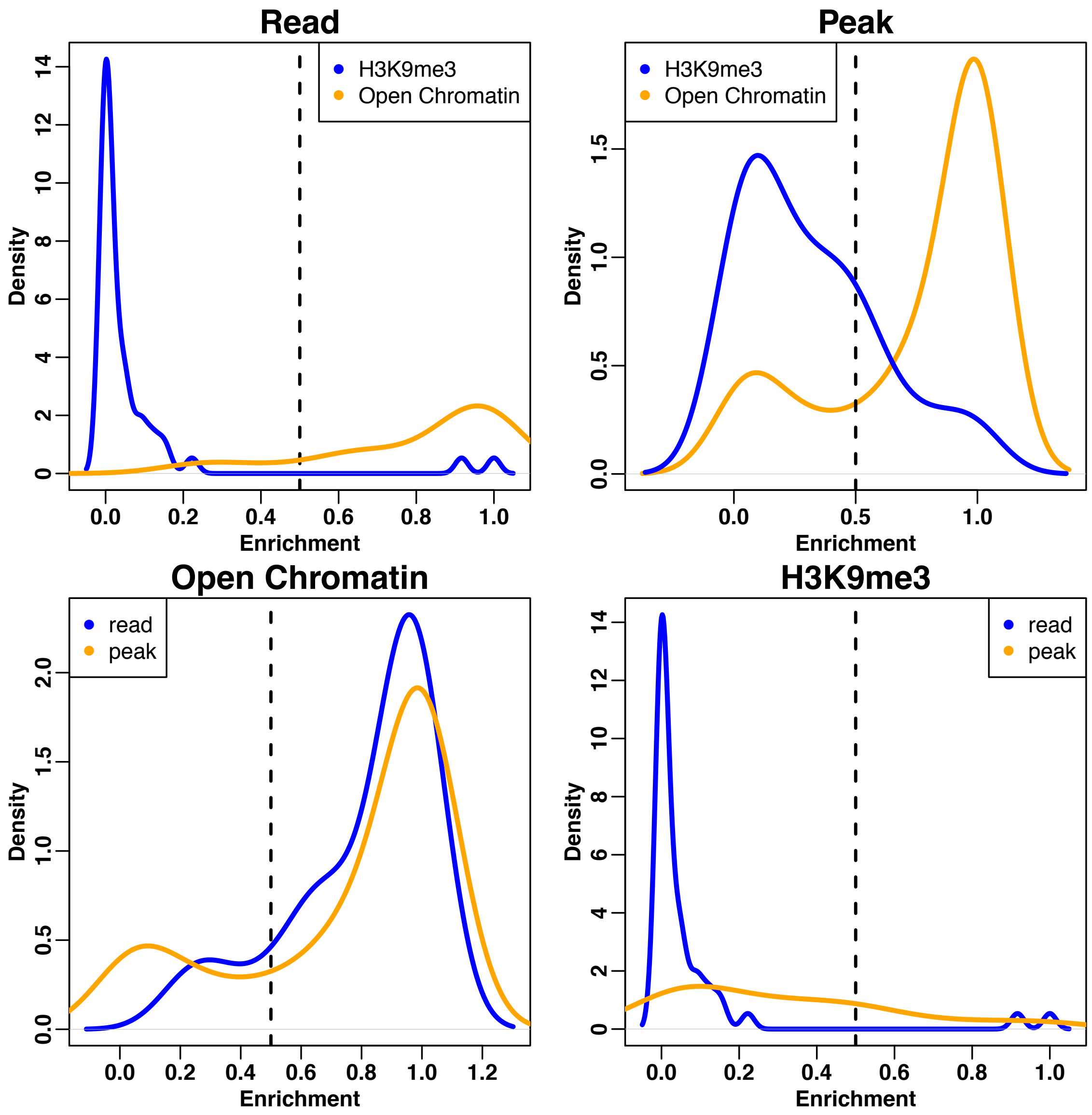
Read
H3K9me3
Open Chromatin
Density
Enrichment
Peak
H3K9me3
Open Chromatin
Density
Enrichment
Open Chromatin
read
peak
Density
Enrichment
H3K9me3
read
peak
Density
Enrichment

Figure S10-A

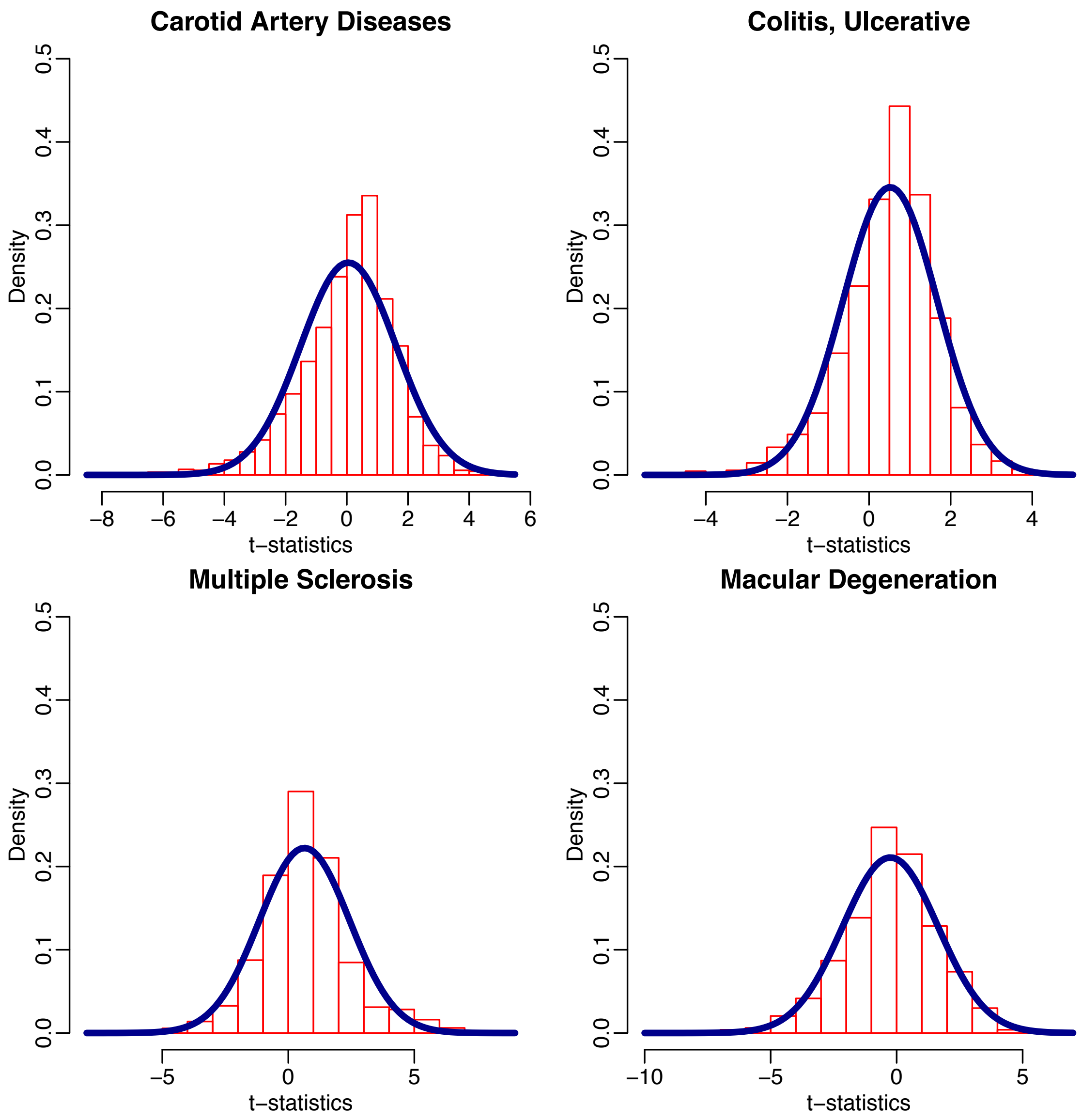

Carotid Artery Diseases
Colitis, Ulcerative
Multiple Sclerosis
Macular Degeneration
Density
t−statistics
0.0
0.1
0.2
0.3
0.4
0.5
−8
−6
−4
−2
0
2
4
6
−5
5
−10

Figure S10-B

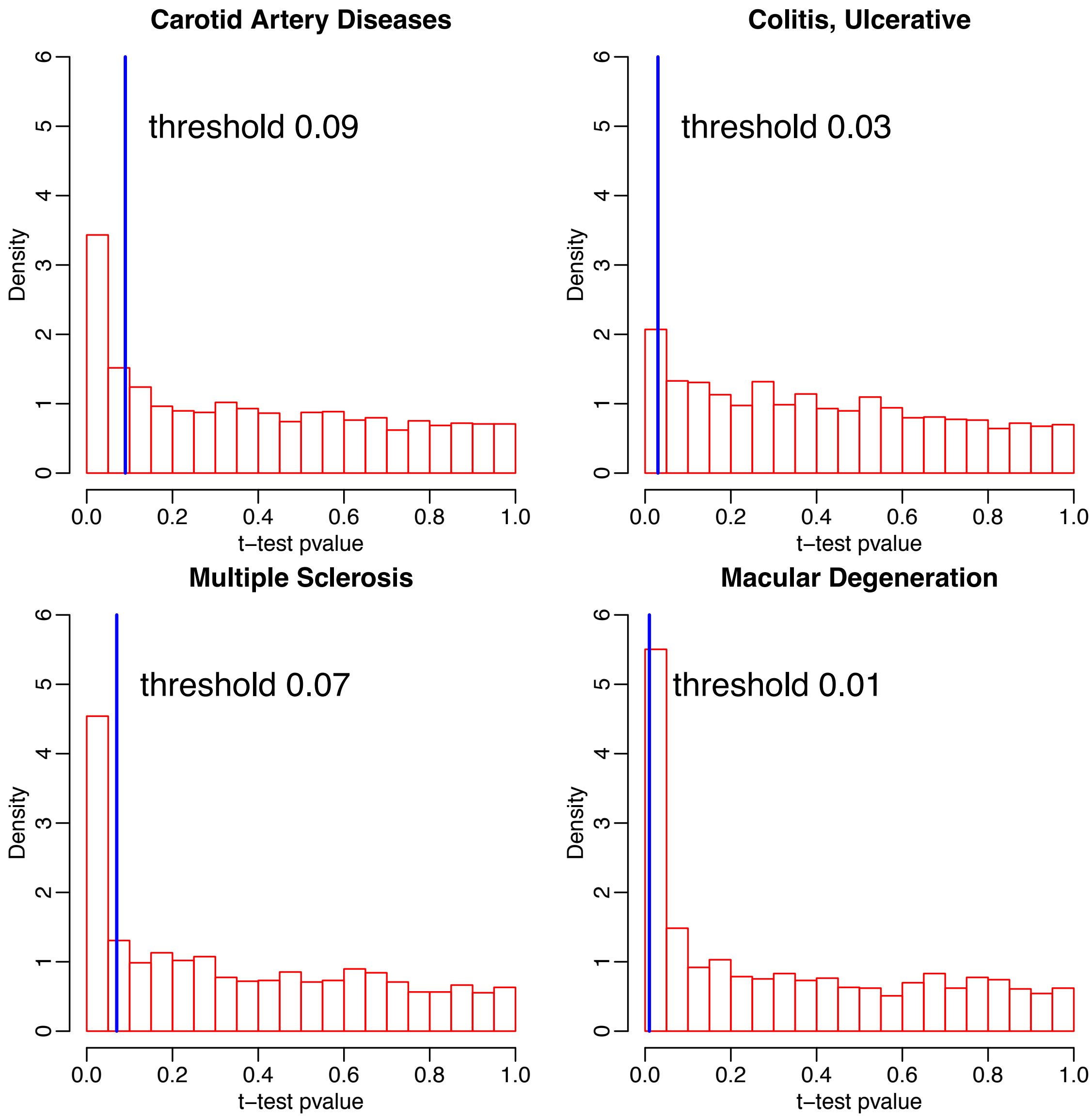

Carotid Artery Diseases
threshold 0.09
Colitis, Ulcerative
threshold 0.03
Multiple Sclerosis
threshold 0.07
Macular Degeneration
threshold 0.01
Density
t−test pvalue
0.0
0.2
0.4
0.6
0.8
1.0
0
1
2
3
4
5
6

Figure S10-C

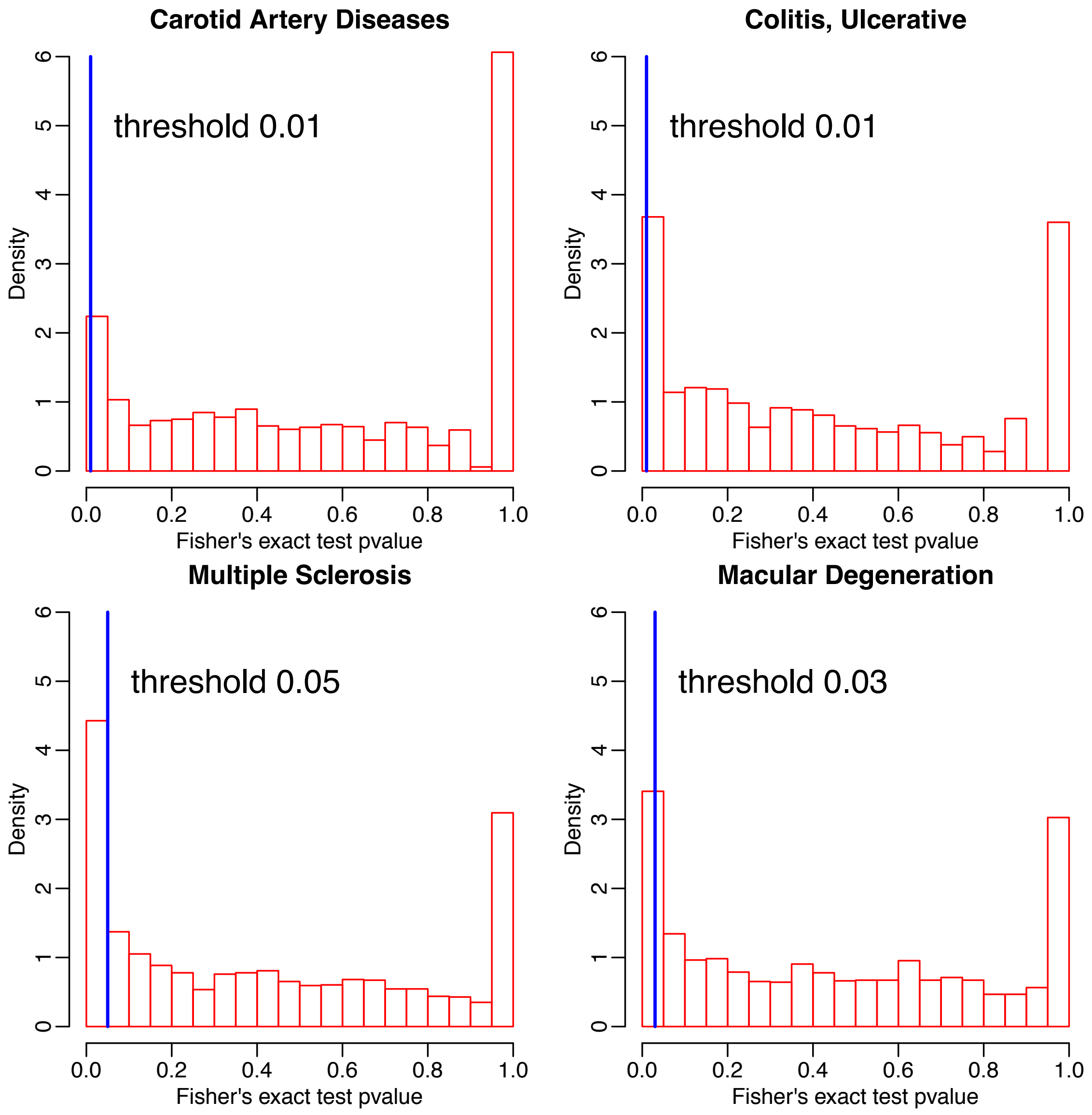

Carotid Artery Diseases
threshold 0.01
Density
Fisher's exact test pvalue
0.0
0.2
0.4
0.6
0.8
1.0
0
1
2
3
4
5
6
Colitis, Ulcerative
threshold 0.01
Multiple Sclerosis
threshold 0.05
Macular Degeneration
threshold 0.03

Figure S11-A

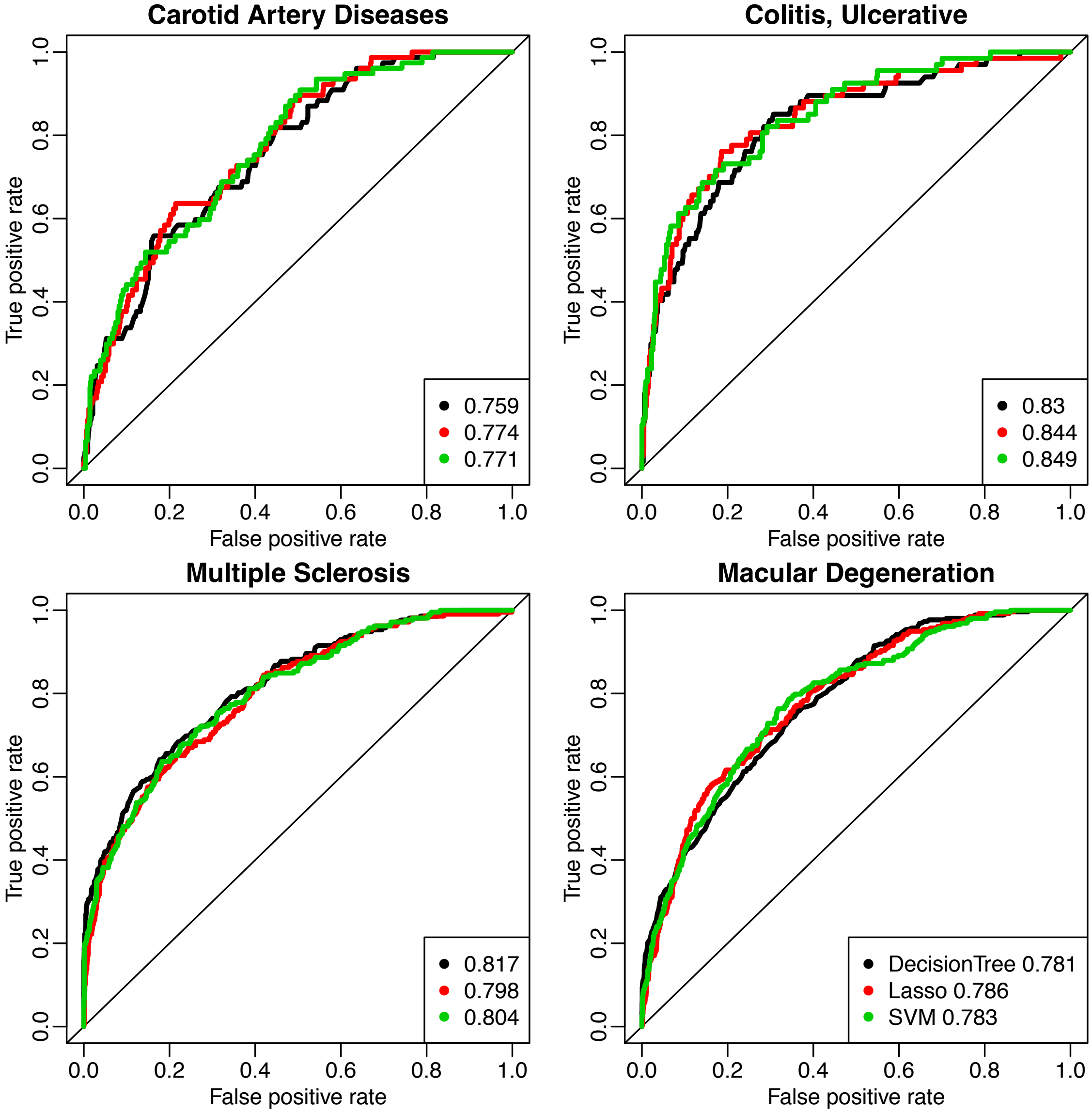

Carotid Artery Diseases
True positive rate
False positive rate
0.0
0.2
0.4
0.6
0.8
1.0
0.759
0.774
0.771
Colitis, Ulcerative
0.83
0.844
0.849
Multiple Sclerosis
0.817
0.798
0.804
Macular Degeneration
DecisionTree 0.781
Lasso 0.786
SVM 0.783

FigureS11-B

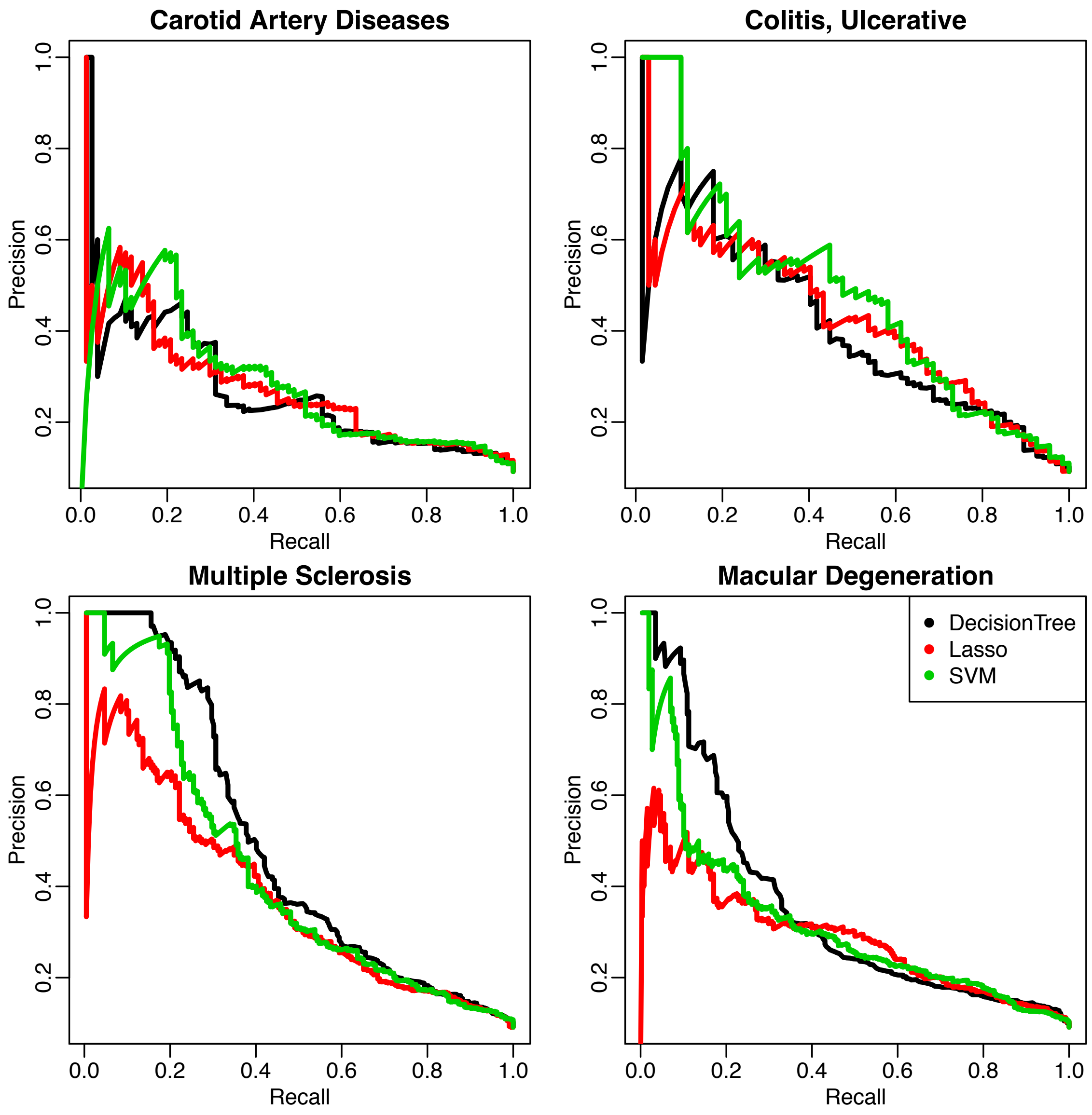

Carotid Artery Diseases
Colitis, Ulcerative
Multiple Sclerosis
Macular Degeneration
Precision
Recall
0.0
0.2
0.4
0.6
0.8
1.0
DecisionTree
Lasso
SVM
